# Machine learning for diagnosing non-ST-segment elevation myocardial infarction: a derivation and validation study

**DOI:** 10.1016/j.eclinm.2026.104055

**Published:** 2026-07-09

**Authors:** Arnaud Champetier, Pedro Lopez-Ayala, Christoph Reich, Jasper Boeddinghaus, Philippe Cattin, Luca Koechlin, Òscar Miró, Michael Christ, Dagmar Iris Keller, Francisco Javier Martín-Sánchez, Beata Morawiec, Jiri Parenica, Emel Kaplan, Paolo Bima, Lourdes Herraiz Recuenco, Gabrielle Huré, Dani Herzka, Karin Wildi, Luca Crisanti, Koray Durak, Julia Sophia Schaffer, Tobias Zimmermann, Felix Mahfoud, Bertil Lindahl, Evangelos Giannitsis, Ivo Strebel, Christian Mueller, APACE, Carlos Spagnuolo, Jonas Glaeser, Thomas Nestelberger, Desiree Wussler, Maria Rubini Gimenez, Danielle Menosi Gualandro, Christian Puelacher, Jeanne du Fay de Lavallaz, Julia Reinhardt, Kathrin Meissner, Katharina Rentsch, Ksenia Slankamenac, Beatriz López, Gemma Martinez-Nadal, Esther Rodriguez Adrada, Arnold von Eckardstein, Damian Kawecki, Piotr Muzyk, Nicolas Geigy, Eliška Potluková, Stephan Steuer, Angelika Hammerer-Lercher, Andreas Buser, TRAPID-AMI Investigators, James McCord, Richard Nowak, Richard Body, Christopher deFilippi, Robert Christenson, Mauro Panteghini, Mario Plebani, Franck Verschuren, John French, Silvia Weiser, Tomas Jernberg, Aitor Alquézar-Arbé, Jordi Ordonez-Llanos

**Affiliations:** a Cardiovascular Research Institute Basel (CRIB) and Department of Cardiology, University Hospital Basel, University of Basel, Basel, Switzerland; b Department of Cardiology, Angiology, and Pneumology, University Hospital Heidelberg, Heidelberg, Germany; c Department of Biomedical Engineering, University of Basel, Basel, Switzerland; d Department of Cardiac Surgery, University Hospital Zurich, University of Zurich, Switzerland; e Emergency Department, Hospital Clínic, Barcelona, Spain; f Emergency Department, Kantonsspital Luzern, Switzerland; g Emergency Department, University Hospital Zurich, Zurich, Switzerland; h Servicio de Urgencias, Hospital Clínico San Carlos, Madrid, Spain; i 2nd Department of Cardiology, School of Medicine with the Division of Dentistry in Zabrze, Medical University of Katowice, Poland; j Department of Cardiology, University Hospital Brno, Czech Republic; k Department of Cardiology, Uppsala University Hospital, Sweden

**Keywords:** Machine learning, Myocardial infarction, External validation, Troponin

## Abstract

**Background:**

Previously developed machine-learning (ML)-based decision support tools for patients presenting with suspected non-ST-segment elevation myocardial infarction (NSTEMI) remain proprietary, limiting public accessibility and clinical adoption.

**Methods:**

To address this limitation, we used two international prospective multicentre diagnostic studies for model derivation and internal validation, and one large prospective European study for external validation, evaluating two open-access ML-based models. The derivation and internal validation cohort comprised 8763 patients (34% women) enrolled across 13 and 12 sites, respectively, in Switzerland, Spain, the Czech Republic, Poland, Belgium, Germany, the UK, Italy and the USA between April 2006 and September 2020, and between August 2011 and June 2013. The external validation cohort included 4882 patients (41% women) from Germany. Patients were excluded if they had a ST-segment elevation myocardial infarction, unclear final diagnosis, renal failure or an absent 12-lead electrocardiogram. A single-high-sensitivity cardiac troponin (hs-cTn) model incorporated information available at emergency department presentation, while a serial-hs-cTn model additionally utilised the second hs-cTn measurement and the time interval between samples. The final diagnosis of NSTEMI was centrally adjudicated by two independent cardiologists in all studies. The diagnostic performance of both models was compared with the European Society of Cardiology (ESC) hs-cTn-0/1 h-algorithm. This study is registered with ClinicalTrials.gov, numbers NCT00470587 and NCT03111862.

**Findings:**

The single-hs-cTn- and serial-hs-cTn model demonstrated excellent discrimination in internal validation, and external validation, with an area under the receiver-operating-characteristic curve of 0.94 [0.92–0.95], and 0.91 [0.90–0.92], and 0.96 [0.96–0.97], and 0.96 [0.95–0.97], respectively. Calibration was good across all datasets. Compared to the ESC-0/1 h algorithm, FAST-NSTEMI provided comparable safety metrics while triaging more patients to rule-out or rule-in. Triage efficacy improved substantially with the single-hs-cTn model (internal validation 52.4% versus 29.8%, external validation 32.1% versus 14.9%) and modestly with the serial-hs-cTn model (internal validation 80.8% versus 76.9%, external validation 77.1% versus 72.8%, all p < 0.01).

**Interpretation:**

The FAST-NSTEMI ML-models offer excellent discrimination, good calibration, high safety, and improved triage efficacy compared to the ESC 0/1 h-algorithm. Further external validation or prospective implementations are warranted to confirm the generalisability of the findings and the clinical utility of the models.

**Funding:**

10.13039/100000001Swiss National Science Foundation and 10.13039/100002129Swiss Heart Foundation.

**Research in context**

**Evidence before this study**

We continuously monitored the literature on machine-learning–based diagnostic decision support tools for patients presenting with suspected non-ST-segment elevation myocardial infarction (NSTEMI) before and throughout the development of this study. This included PubMed search and screening of publications between Jan 1, 2018, and Jan 1, 2026. Search terms combined concepts related to machine learning and artificial intelligence with NSTEMI, myocardial infarction, troponin, triage, emergency department settings, and external validation. We excluded studies focused on prognosis, those conducted in non-emergency care settings, and studies addressing diagnostic questions other than NSTEMI.

Previously developed machine-learning-based decision support tools for patients presenting with suspected NSTEMI have remained proprietary, restricting public access and thereby broader clinical application. Additionally, many models rely on numerous predictors or are based on limited datasets, increasing the risk of overfitting and compromising generalisability.

**Added value of this study**

To our knowledge, this is the first study to develop both single and serial high-sensitivity cardiac troponin (hs-cTn) machine learning models using data from two international, prospective, multicentre studies, with the models being made openly available. These models were independently validated in a separate single-centre cohort. The FAST-NSTEMI models use just 6–8 variables and allow for flexible timing of the serial hs-cTn measurement. Their diagnostic performance in external validation was good and comparable to that observed in internal validation. Furthermore, its performance was benchmarked against the European Society of Cardiology (ESC) hs-cTn 0/1 h algorithm, demonstrating improved triage efficiency.

**Implications of all the available evidence**

FAST-NSTEMI provides a transparent, accessible and comprehensive machine learning–based framework for the early triage of patients with suspected NSTEMI. The framework demonstrated robust diagnostic performance and appears to offer advantages over the current hs-cTn 0/1 h algorithm. Future work should focus on further external validation and prospective implementation, aided by the publicly available models.

## Introduction

Early and accurate diagnosis of acute myocardial infarction (AMI) is essential to initiate timely, evidence-based treatment. The implementation of high-sensitivity cardiac troponin (hs-cTn) assays into clinical practice has significantly improved the early detection of AMI and enabled the development of several effective hs-cTn-based diagnostic algorithms.^1^, ^2^, ^3^, ^4^, ^5^

Despite these advances, current guideline-recommended hs-cTn-based diagnostic algorithms have important limitations that affect their feasibility, acceptance, efficacy, and accuracy in clinical practice.^1^^,^^4^^,^^5^ These include 1) using binary hs-cTn thresholds instead of continuous values; 2) neglecting key hs-cTn confounders such as age; 3) exclusion of clinical characteristics and 12-lead electrocardiogram (ECG) data; and 4) use of fixed rather than flexible time intervals to the second hs-cTn measurement. To overcome some of these limitations, several machine-learning (ML) -models have been developed.^6^, ^7^, ^8^, ^9^, ^10^, ^11^ However, they omit key hs-cTn confounders, use large numbers of variables that reduce practicality and feasibility in busy emergency departments (ED), or are developed based on small sample-size cohorts, compromising generalisability.^12^ Moreover, all these ML-based tools remain inaccessible or proprietary, hindering academic evaluation and thereby reducing trust and clinical adoption.

To address these unmet needs, we aimed to derive and externally validate an open-access sequential ML framework for a diagnosis of non-ST-segment elevation MI (NSTEMI, “FAST-NSTEMI”), applying three key innovations to enhance clinical feasibility and diagnostic performance: i) use of a concise set of variables; ii) individualised hs-cTn thresholds, accounting for known confounders; and iii) incorporating flexible, real-world time intervals for serial hs-cTn measurement. To reflect real-world clinical workflows, we developed two ML-based models: a single-hs-cTn model using only data available at ED presentation, and a serial-hs-cTn model incorporating the second hs-cTn value and the time interval between measurements. The performance of both models was compared against the current guideline-recommended standard-of-care, the European Society of Cardiology (ESC) hs-cTn-0/1 h-algorithm.^5^^,^^13^

## Methods

### Study design and population

Two prospective international, multicentre diagnostic studies recruiting adult patients presenting to the ED with acute chest discomfort suggestive of AMI and centrally adjudicating the final diagnosis were used for the derivation of the FAST-NSTEMI, which comprises both a single hs-cTnT model and a serial hs-cTnT model: APACE (Advantageous Predictors of Acute Coronary Syndromes Evaluation, www.clinicaltrials.gov; NCT00470587)^6^^,^^8^^,^^14^^,^^15^ and TRAPID-AMI^16^^,^^17^ (The High Sensitivity Cardiac Troponin T Assay for Rapid Rule-out of Acute Myocardial Infarction).

Given the poor harmonisation among the different hs-cTnT/I-assays, this analysis focused on the currently best-validated and most widely used hs-cTnT/I-assay, i.e., hs-cTnT-gen5 (Roche Diagnostics, Supplemental Methods).^18^ Patients were eligible for this analysis if they had at least one hs-cTnT measurement. Patients were excluded, if they had: (i) ST-segment-elevation MI; (ii) unclear final diagnosis even after final adjudication and at least one hs-cTnT concentration above the upper reference limit, thereby possibly indicating AMI; (iii) dialysis-dependent end-stage renal disease; (iv) no 12-lead ECG; and (v) no reported time since chest pain onset (CPO) at ED presentation. Patients without a second hs-cTnT sample were excluded from the derivation and validation dataset for the serial-hs-cTnT model (Supplemental Figure S1).

### Ethics

The studies were carried out according to the principles of the Declaration of Helsinki and approved by each local ethics committee and centrally approved in Basel by the Ethikkommission Nordwest-und Zentralschweiz (EKNZ) (EKNZ-nr. for APACE: 280/05; TRAPID-AMI: 230/11). Written informed consent was obtained from all patients. Reporting follows the TRIPOD + AI (Transparent Reporting of a Multivariable Prediction Model for Individual Prognosis or Diagnosis—Artificial Intelligence) statement (Supplemental File).^19^

### Central adjudication of the final diagnosis

Adjudication of the final diagnosis was performed centrally by two independent cardiologists according to current guidelines and the 4th Universal Definition of Myocardial Infarction (UDMI).^6^^,^^8^^,^^20^ In situations of disagreement, a third cardiologist was involved. All available medical records, including cardiac imaging and two sets of serial hs-cTnI/T measurements (one clinical and one from study blood), were used. Mandatory variables for the central adjudication of NSTEMI and therefore also this analysis were defined a priori and included patient demographics, the 12-lead ECG, and hs-cTnT (Supplemental Figure S1). Accordingly, there were no missing data for these variables. However, as the choice of the work-up strategy including cardiac imaging was determined by the treating physician, not all cardiac imaging procedures were available in all patients.^6^^,^^8^^,^^14^^,^^21^ The agreement rate for NSTEMI in the APACE cohort was 82.2% and unavailable in TRAPID-AMI. A second central adjudication using the sex-specific instead of the regulatory-approved uniform upper reference limit (URL) was used in a sensitivity analysis.

### Outcome definition

The outcome was a centrally adjudicated diagnosis of NSTEMI (type 1 and 2).

### Variable selection and variable creation

Variables for the models were selected based on prior studies identifying predictors of NSTEMI.^4^^,^^5^^,^^20^^,^^22^, ^23^, ^24^ The single-hs-cTnT model was derived using hs-cTnT at ED presentation, age, sex, time since CPO, creatinine concentration, and four ECG criteria (normal, T-wave inversion, ST-segment depression, bundle branch block (BBB) or pacemaker-ECG). The serial-hs-cTnT model also included a second hs-cTnT concentration and the time interval between the first (0 h) and the second blood draw. The change in hs-cTnT over time was calculated as the difference in hs-cTnT concentrations (ng/l) divided by the time interval (min) between the blood draws. As in some patients time since CPO cannot be reliably assessed, we derived an alternative model omitting this variable in a sensitivity analysis.

### Upsampling

Since ML requires large amounts of data,^12^^,^^19^ the derivation dataset for the single-hs-cTnT model was maximised by treating patients with serial hs-cTnT measurements as multiple presentations. Similarly, for the derivation of the serial-hs-cTnT model, all possible hs-cTnT pairs were considered as separate presentations (0/1 h, 0/2 h, 0/3 h, 1/2 h, 1/3 h, 2/3 h). Although this approach may introduce dependence between observations, it reflects clinically plausible variation in presentation timing, whereby later measurements may constitute a baseline hs-cTnT assessment. This strategy enables the model to learn from realistic temporal presentation patterns. For the internal validation, only the 0 h hs-cTnT and the first serial hs-cTnT measurement were used. The comparison with the ESC 0/1 h algorithm was performed only using patients with 0 h and 1 h hs-cTnT measurements.

### Model derivation and internal validation

The pooled (APACE + TRAPID-AMI) dataset was divided into training (80%) and testing (20%) sets for internal validation. The extensively validated XGBoost algorithm was used to develop the FAST-NSTEMI ML framework.^25^ XGBoost is an ML algorithm that boosts predictive accuracy by combining multiple weak decision trees. It constructs these trees sequentially, with each new tree designed to correct the errors of the prior ones. This iterative process continues until a predefined number of trees is reached. The scores (log odds) from each decision tree are summed and converted to a probability using a sigmoid function (Supplemental Methods).^25^ Hyperparameter tuning was performed on the training set using nested cross-validation and sampling from predefined distributions, with the Brier score as the selection criterion. Detailed methods for model derivation are provided in the Supplemental Methods.

### ESC hs-cTnT-0/1 h-algorithm and diagnostic scores

The ESC 0/1 h-algorithm was applied as recommended by current guidelines (Supplemental Material).^5^ The diagnostic performance of the recalibrated TIMI and HEART scores were compared with FAST-NSTEMI.^26^

### External validation: RAPID-CPU

External validation was performed in the independent single-centre prospective RAPID-CPU (www.clinicaltrials.gov; NCT03111862) study, recruiting consecutive patients presenting with suspected AMI to the ED of the Heidelberg University Hospital (Supplemental Methods).^27^^,^^28^

### Statistical analysis

Categorical variables were reported as count (percentage), and continuous variables as median (25th–75th percentiles [Q1–Q3]). The 95% confidence intervals were calculated using Wilson's method if not otherwise specified. To appraise the diagnostic performance of FAST-NSTEMI, its discrimination and calibration were evaluated.^29^ Model discrimination was quantified using the area under the receiver operating characteristic curve (AUC). Confidence intervals for the AUC were estimated using the DeLong et al. method if not specified otherwise.^30^ If Delong's independence of observations assumption were violated, confidence intervals were computed using the non-parametric bootstrap method.^31^ To assess agreement between observed and predicted probabilities, calibration curves with logistic regression and Loess smoothing were constructed.^32^^,^^33^ The p-value for the difference between correlated proportions was assessed using the McNemar method.^34^

Triage thresholds (rule-out and rule-in of NSTEMI) were derived based on the continuous probability output of each model. Target sensitivity and negative predictive value (NPV) for rule-out were 99% and 99.5%, respectively. Target specificity and positive predictive value (PPV) for rule-in were 95% and 70%, respectively. Triage thresholds were derived in the derivation cohort and chosen conservatively, anticipating a possible drop in performance in external validation. These thresholds were also used to compare FAST-NSTEMI versus the guideline-recommended standard-of-care ESC hs-cTnT-0/1 h algorithm; however, this comparison may inherently favour the developed model.

Four sensitivity analyses were conducted. First, to mitigate potential recall bias associated with the time since CPO, an alternative version of FAST-NSTEMI was derived, omitting this variable. This analysis was also performed in the external validation cohort. Second, we evaluated the robustness of the model when applying sex-specific URLs instead of a uniform URL for the adjudication of NSTEMI (post-hoc). Third, we assessed the diagnostic performance of the FAST-NSTEMI using the combination of 0- and 2-h value of hs-cTnT. Fourth, we assessed the diagnostic performance of the FAST-NSTEMI when used with an alternative hs-cTn assay (post-hoc): the hs-cTnI Architect (ARCHITECT STAT hs-cTnI, Abbott Laboratories) (Supplemental Methods).

All statistical analyses were performed using R 4.2.1 (R Foundation for Statistical Computing, Vienna, Austria). The R packages are listed in the Supplemental Methods.

### SHAP

SHapley Additive exPlanations (SHAP) values were employed to investigate the contribution of each feature in FAST-NSTEMI to model performance (Supplemental Methods).

### Role of the funding source

The funders had no involvement in study design, data collection, data analyses, data interpretation, or the writing of the report.

## Results

### Patient characteristics

A total of 9549 patients (8267 from APACE and 1282 from TRAPID-AMI) had at least one 0 h hs-cTnT measurement available. The median age was 61 [49–74] years, 3265 (34%) were female, and the median time since CPO was 5 [2, 15] hours (Table 1). After applying the exclusion criteria, 8763 patients were eligible for the derivation (n = 7011) and internal validation (n = 1752) of the single-hs-cTnT model, and 7878 patients were eligible for the derivation (n = 6465) and internal validation (n = 1413) of the serial-hs-cTnT model. After upsampling, the cohort for the single-hs-cTnT model reached 24,261 patients: 19,412 (80%) for derivation, and 4849 (20%) for internal validation (Supplemental Figure S2, Supplemental Table S1). The cohort for the serial-hs-cTnT model reached 25,530 patients: 20,442 (80%) for derivation, and 5088 (20%) for internal validation (Supplemental Figure S3, Supplemental Table S1). Patients’ characteristics per centre is shown in the Supplemental Material (Supplemental Tables S2 and S3).

**Table 1:** Baseline characteristics of the development and external validation cohorts.

Cohorts	Derivation & Internal validation (n = 9549)	External validation (n = 5220)
Demographics		
Age, y (median [IQR])	61.0 [49.0, 74.0]	65.0 [52.0, 77.0]
Female, n (%)	3265 (34)	2152 (41)
Time since CPO (continuous)	5 [2, 15]	NA
Time since CPO (categorised)		
0–3 h	2982 (31)	1030 (20)
3–6 h	2110 (22)	454 (9)
6 h+	4362 (46)	2398 (46)
NA	95 (1)	1338 (26)
Cardiovascular risk factors, n (%)		
Hypertension	5714 (60)	3364 (64)
Hypercholesterolaemia	4000 (48)	2267 (43)
Diabetes mellitus	1737 (18)	1046 (20)
Current smoker	2814 (30)	1072 (21)
History, n (%)		
Previous CAD	3129 (33)	1819 (35)
Previous MI	2179 (23)	888 (17)
Previous revascularisation	2337 (24)	1427 (27)
Previous stroke	429 (5)	NA
Laboratory findings, median (IQR)		
eGFR, ml/min/1.73 m^2^	86 [69, 101]	84 [65, 98]
Medications at presentation, n (%)		
Antiplatelet therapy	3261 (34)	NA
Oral anticoagulation	998 (10)	NA
β-blocker	3203 (34)	NA
Statin	2843 (34)	NA
ACEIs/ARBs	3608 (38)	NA
12-lead ECG findings, n (%)		
Aberration (BBB, pacemaker)	559 (6)	620 (12)
ST depression	858 (9)	362 (7)
T-Wave inversion	890 (9)	1233 (24)
None	7138 (76)	NA
Cardiac troponin, median (IQR)		
Hs-cTnT 0 h	8 [4, 22]	9 [5, 24]
Hs-cTnT 1 h	8 [4, 22]	–
Hs-cTnT 2 h	9 [4, 24]	–
Hs-cTnT 3 h	11 [5, 33]	–
First serial Hs-cTnT	8 [4, 23]	9 [5, 18]
Final diagnosis, n (%)		
NSTEMI	1596 (17)	673 (13)

IQR, interquartile range; ECG, Electrocardiogram; CPO, chest pain onset; CAD, coronary artery disease; MI, myocardial infarction; eGFR, estimated glomerular filtration rate; ACEIs/ARBs, Angiotensin-converting enzyme inhibitors or angiotensin receptor blocker; BBB, Bundle branch block; Hs-cTn, high-sensitivity-cardiac troponin; NSTEMI, Non-ST-segment elevation myocardial infarction. Hypercholesterolaemia and statin use were not recorded in TRAPID-AMI; percentages are based on APACE only, n = 8267.

In the independent external validation cohort, 5220 patients, median age 65 [52–77] years, 41% women, were recruited. After applying the exclusion criteria, 4882 and 1915 patients were eligible for the external validation of the single and serial-hs-cTnT models, respectively (Supplemental Figure S4).

### Explainability

**Fig. 1:**
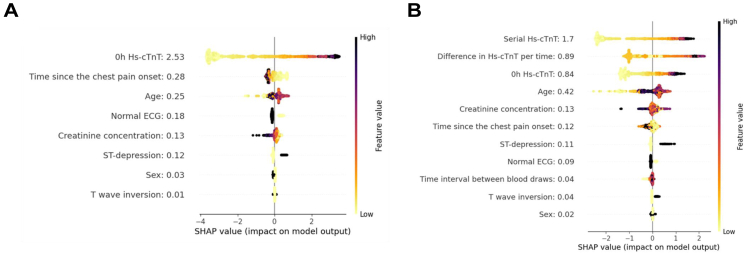
**Mean absolute SHapley Additive exPlanations (SHAP) values (average impact on the model output magnitude) with the impact of each feature on each model output.** Using (A) the single-hs-cTnT model and (B) the serial-hs-cTnT model. The summary plots show the effect of each feature on individual predictions, represented as small points. A dense cluster of points indicates that many patients experienced a similar impact due to the value of that feature, i.e. in the first model, very low troponin levels were frequently associated with a strong negative impact on the log odds (A) and in the second model, very high serial troponin levels were frequently associated with strong positive impact on the log odds (B). The computation of the magnitudes and the impact of each variable on a single prediction are described in the Supplemental Material. hs-cTnT, high-sensitivity cardiac troponin T; ECG, Electrocardiogram.

In the single-hs-cTnT model, NSTEMI risk was associated with hs-cTnT concentration at ED presentation, time since chest pain onset and age. In the serial-hs-cTnT model, change in hs-cTnT over time provided additional information (Fig. 1).

### Derivation cohort

#### Discrimination and calibration

The discriminative performance of FAST-NSTEMI for detecting NSTEMI in the upsampled derivation cohort was very good, with an AUC of 0.96 (95% CI: 0.96–0.97, Supplemental Figure S5A) for the single-hs-cTnT model and 0.98 (95% CI: 0.98–0.98, Supplemental Figure S5C) for the serial-hs-cTnT model. Overall, calibration was good for the single-hs-cTnT model with an intercept of −0.05 [95% CI: −0.11 to 0.01] and a slope of 1.11 [95% CI: 1.08–1.15] (Supplemental Figure S5B). Similar results were found using the serial-hs-cTnT model with an intercept of 0.06 [95% CI: 0.00–0.13] and a slope of 1.26 [95% CI: 1.21–1.30] (Supplemental Figure S5D). Discriminative performance in the non-upsampled cohort remained consistent with the upsampled cohort (Supplemental Figure S5E–H).

### Internal validation

#### Discrimination and calibration

**Fig. 2:**
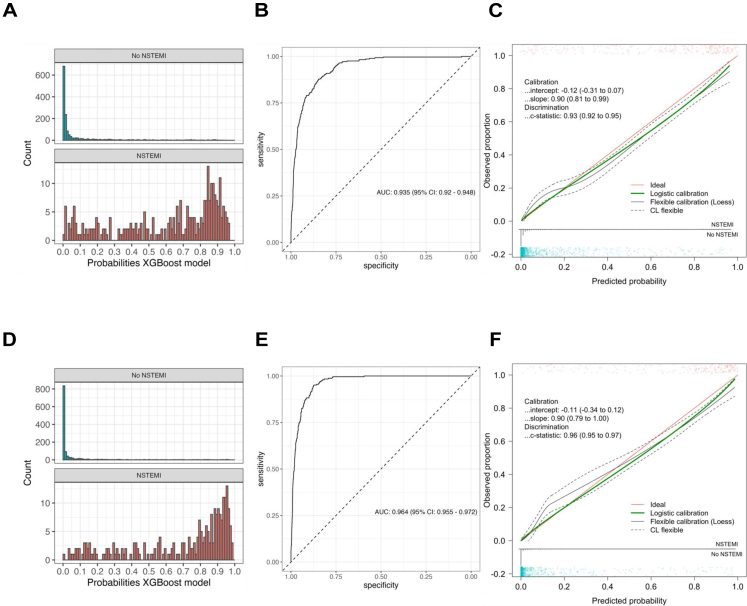
**Diagnostic performance of the FAST-NSTEMI in the internal validation cohort.** (A) Distribution of predicted probabilities as calculated by FAST-NSTEMI using a single hs-cTnT measurement; (B) Receiver-operating-characteristic curve showing the discrimination of FAST-NSTEMI for NSTEMI using a single hs-cTnT measurement; (C) Calibration curve of the FAST-NSTEMI algorithm using a single hs-cTnT measurement, assessing the agreement between predicted probabilities and observed proportion. The red line represents optimal calibration, the black solid line represents the calibration curve derived using loess smoothing while the green line represents the calibration curve derived using logistic regression, and the black dashed line represents the 95% confidence interval of the calibration curve. (D) Distribution of predicted probabilities as calculated by FAST-NSTEMI using two serial hs-cTnT measurements; (E) Receiver-operating-characteristic curve showing the discrimination of FAST-NSTEMI for NSTEMI using two serial hs-cTnT measurements; (F) Calibration curve of the FAST-NSTEMI algorithm using two serial hs-cTnT measurements, assessing the agreement between predicted probabilities and observed proportion. hs-cTnT, high-sensitivity cardiac troponin T; AUC, Area under the receiver operating characteristic curve; NSTEMI, non-ST-segment-elevation myocardial infarction; CL flexible, confidence limits of the flexible calibration; c-statistic = AUC.

The distribution of the probabilities computed by FAST-NSTEMI ranged from 0 to 97% using the single-hs-cTnT model and from 0 to 99% using the serial-hs-cTnT model. The discriminative performance of FAST-NSTEMI for detecting NSTEMI in the internal validation cohort was very good with an AUC of 0.94 (95% CI: 0.92–0.95, Fig. 2B) for the single-hs-cTnT model and 0.96 (95% CI: 0.96–0.97, Fig. 2E) for the serial-hs-cTnT model. Overall, calibration was good for the single-hs-cTnT model with an intercept of −0.12 [95% CI: −0.31 to 0.07] and a slope of 0.90 [95% CI: 0.81–0.99], showing little overestimation and overfitting (Fig. 2C). Similar results were found using the serial-hs-cTnT model with an intercept of −0.11 [95% CI: −0.34 to 0.12] and a slope of 0.90 [95% CI: 0.79–1.00] (Fig. 2F). In the subgroup analysis, using the single-hs-cTnT model, discrimination was lower in older patients (age >65 years) and in early presenters (Supplemental Figure S6A). Using the serial-hs-cTnT model, discrimination was again lower in older patients and patients with low estimated glomerular filtration rate (eGFR) (Supplemental Figure S6B).

#### Triage thresholds

Triage thresholds showed good performance in the derivation and internal validation cohort (Supplemental Results, Supplemental Table S4, Table 2 and Supplemental Figure S7).

**Table 2:** Diagnostic thresholds of FAST-NSTEMI in the internal validation cohort.

Threshold (%)	Rule-in thresholds and metrics					
	Single-hs-cTnT model			Serial-hs-cTnT model		
	Specificity	PPV	Proportion RI (%)	Specificity	PPV	Proportion RI (%)
50	0.9495	0.7186	15.01	0.9528	0.7541	16.08
55	0.9557	0.7379	14.16	0.9599	0.7792	15.23
60	0.9611	0.7564	13.36	0.9638	0.7870	14.24
65	0.9652	0.7628	12.27	0.9670	0.7951	13.51
70	0.9700	0.7684	10.84	0.9725	0.8187	12.72
75	0.9795	0.8113	9.08	0.9748	0.8222	11.87
80	0.9836	0.8273	7.93	0.9803	0.8397	10.28

Threshold (%)	Rule-out thresholds and metrics					
	Single-hs-cTnT model			Serial-hs-cTnT model		
	Sensitivity	NPV	Proportion RO (%)	Sensitivity	NPV	Proportion RO (%)
1.0	0.9965	0.9985	38.93	0.9959	0.9988	55.11
1.5	0.9895	0.9963	46.86	0.9959	0.9989	59.00
2.0	0.9755	0.9924	52.80	0.9959	0.9989	61.17
2.5	0.9755	0.9928	55.48	0.9959	0.9990	62.89
3.0	0.9755	0.9931	57.65	0.9959	0.9990	63.94
3.5	0.9720	0.9923	59.30	0.9878	0.9970	65.13
4.0	0.9650	0.9906	60.67	0.9878	0.9970	65.99

NPV, negative predictive value; PPV, positive predictive value; RO, rule-out; RI, rule-in.

#### Direct comparison with the ESC 0/1 h-algorithm and risk scores

**Fig. 3:**
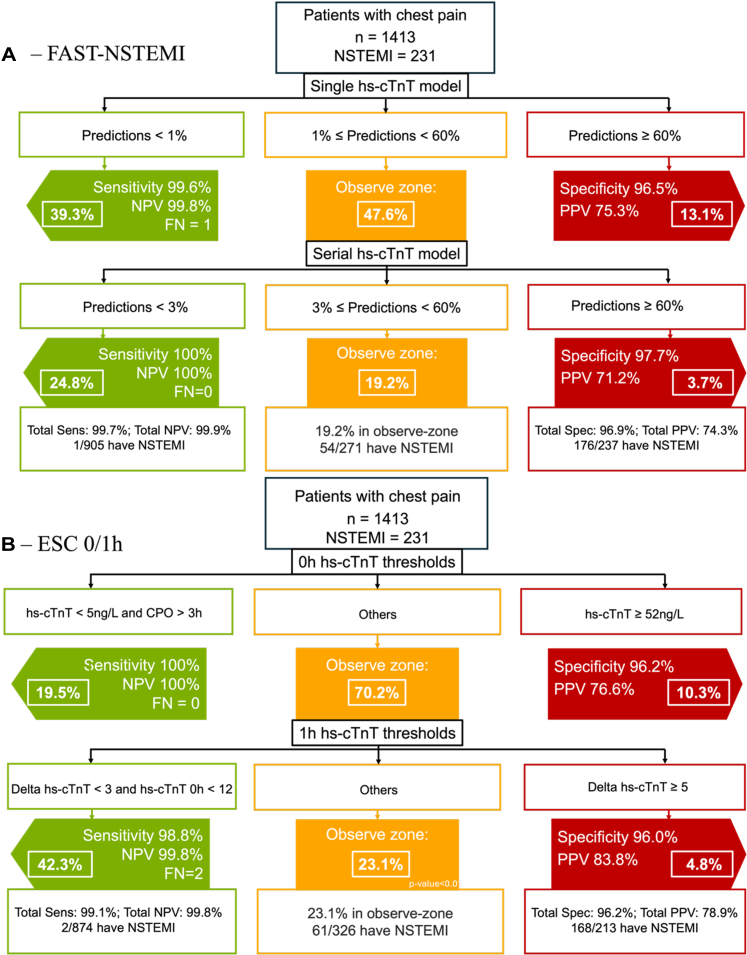
**Performance of the FAST-NSTEMI(A)compared with the ESC 0/1 h-algorithm(B)in the internal validation cohort.** P-values were calculated using the McNemar test to compare the proportion of patients triaged by FAST-NSTEMI versus the ESC 0/1 h-algorithm. ESC, European Society of Cardiology; NSTEMI, non-ST-segment elevation myocardial infarction; NPV, negative predictive value; PPV, positive predictive value; FN, false negative; CPO, time since the chest pain onset; hs-cTnT, high-sensitivity cardiac troponin T; Sens, sensitivity; Spec, specificity.

The FAST-NSTEMI showed superiority in triage efficacy, and it was most notable for the single-hs-cTnT model upon ED presentation triaging 52.4% versus 29.8% of patients by the ESC 0/1 h-algorithm (Fig. 3, Supplemental results). When the serial-hs-cTn model was applied, triage efficacy showed a modest improvement, triaging 80.8% versus 76.9%. The recalibrated TIMI and HEART scores showed lower discrimination performances than the FAST-NSTEMI model in internal validation (Supplemental Figure S8).

#### Sensitivity analyses

Diagnostic performance remained consistent with the main analysis in all the sensitivity analyses (Supplemental Results, Supplemental Tables S5–S7 and Supplemental Figures S9–S12). Diagnostic performance using 0 and 2 h or 0 and 3 h hs-cTnT combinations remained consistent with the main analysis (Supplemental Figure S13).

### External validation

#### Discrimination and calibration

**Fig. 4:**
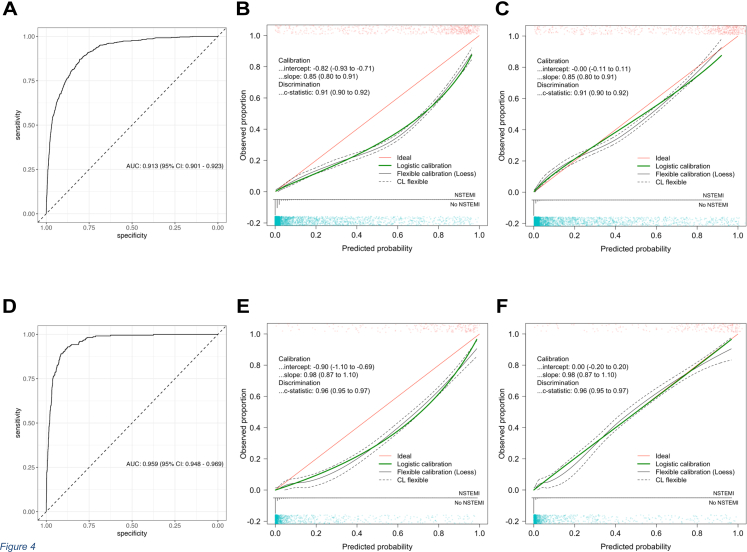
**Diagnostic performance of the FAST-NSTEMI in the external validation cohort.** (A) Receiver-operating-characteristic curve showing the discrimination of FAST-NSTEMI for NSTEMI using a single hs-cTnT measurement; (B) Calibration curve of the FAST-NSTEMI algorithm using a single hs-cTnT measurement, assessing the agreement between predicted probabilities and observed proportion. The red line represents optimal calibration, the black solid line represents the calibration curve derived using loess smoothing while the green line represents the calibration curve derived using logistic regression, and the black dashed line represents the 95% confidence interval of the calibration curve. (C) Calibration curve of the FAST-NSTEMI algorithm using a single hs-cTnT measurement, assessing the agreement between intercept-corrected predicted probabilities and observed proportion. (D) Receiver-operating-characteristic curve showing the discrimination of FAST-NSTEMI for NSTEMI using two serial hs-cTnT measurements; (E) Calibration curve of the FAST-NSTEMI algorithm using two serial hs-cTnT measurements, assessing the agreement between predicted probabilities and observed proportion. (F) Calibration curve of the FAST-NSTEMI algorithm using two serial hs-cTnT measurements, assessing the agreement between intercept-corrected probabilities and observed proportion. Hs-cTnT, High-sensitive cardiac troponin T; AUC, Area under the receiver operating characteristic curve; NSTEMI, non-ST-segment-elevation myocardial infarction; CL flexible, confidence limits of the flexible calibration; c-statistic = AUC.

The discriminative performance of FAST-NSTEMI was very good, with an AUC for predicting NSTEMI using the single hs-cTnT and the serial-hs-cTnT model of 0.91 [95% CI: 0.90–0.92] and 0.96 [95% CI: 0.95–0.97] (Fig. 4A and D), respectively. Calibration showed overprediction using the single hs-cTnT and the serial-hs-cTnT model, respectively, with an intercept of −0.82 [95% CI: −0.93-(−0.71)] and an intercept of −0.90 [95% CI: −1.10-(−0.69)]; the actual risk is lower than the one predicted. However, slopes were moderate-to-good using the single-hs-cTnT model (slope = 0.85 [95% CI: 0.80–0.91]) and using the serial-hs-cTnT model (slope = 0.98 [95% CI: 0.87–1.10]) (Fig. 4B and E). When correcting for the offset in the predictions, the recalibrated curves showed good calibration for both models (Fig. 4C and F).

#### Triage thresholds

Triage thresholds showed good performance in the external validation cohort (Supplemental Results, Supplemental Tables S8 and S9 and Supplemental Figure S14).

#### Direct comparison with the ESC 0/1 h-algorithm

**Fig. 5:**
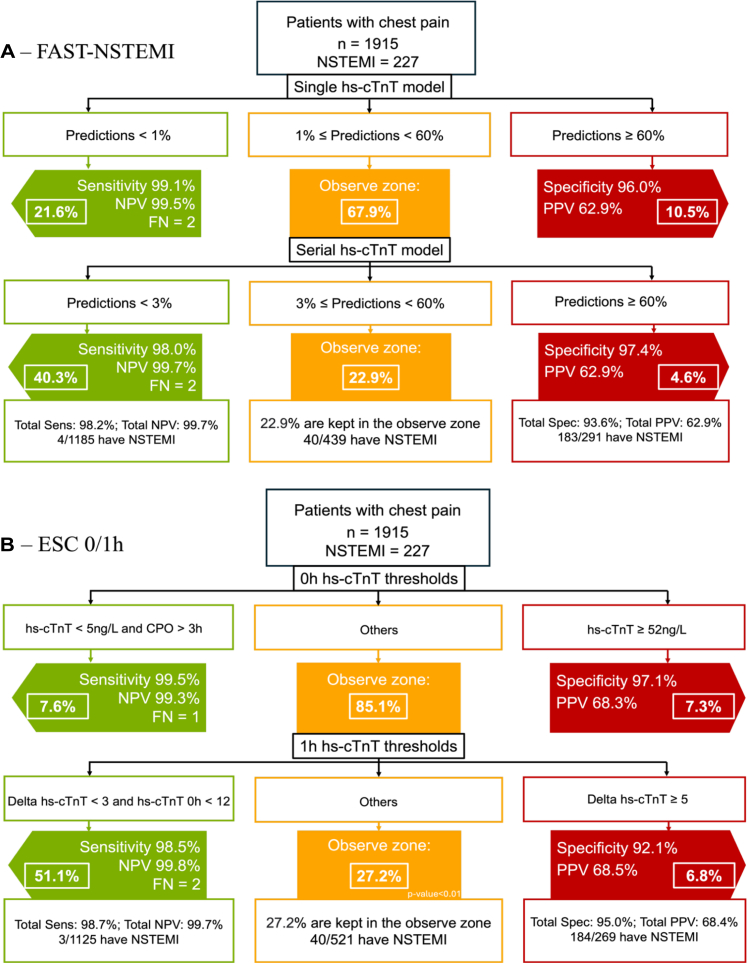
**Performance of the FAST-NSTEMI(A)compared with the ESC 0/1 h-algorithm(B)in the external validation cohort.** P-values were calculated using the McNemar test to compare the proportion of patients triaged by FAST-NSTEMI versus the ESC 0/1 h-algorithm. ESC, European Society of Cardiology; NSTEMI, non-ST-segment elevation myocardial infarction; NPV, negative predictive value; PPV, positive predictive value; FN, false negative; CPO, time since the chest pain onset; hs-cTnT, high-sensitivity cardiac troponin T; Sens, sensitivity; Spec, specificity.

Among 1915 patients eligible for the direct comparison with the ESC 0/1 h-algorithm, 227 (11.9%) had an NSTEMI (Fig. 5). Overall, FAST-NSTEMI classified more patients than the ESC 0/1 h-algorithm to rule out (1185 [61.9%] versus 1125 [58.7%], respectively) and rule-in (291 [15.1%] versus 269 [14.1%], respectively), leaving fewer patients in the observe zone with FAST-NSTEMI (439 [22.9%] versus 521 [27.2%], p < 0.01). FAST-NSTEMI had a similar NPV to the ESC 0/1 h-algorithm: NPV 99.7% [95% CI 99.5–99.9] versus 99.7% [95% CI, 99.5–99.9]. In contrast, sensitivity, specificity and PPV were slightly lower for FAST-NSTEMI versus the ESC 0/1 h-algorithm: sensitivity 98.2% [95% CI, 97.6%–98.8%] versus 98.7% [95% CI, 98.2–99.2]; PPV 62.9% [95% CI, 60.7–65.1] versus 68.4% [95% CI, 66.3–70.5]; specificity 93.6% [95% CI, 92.5–94.7] versus 95.0% [95% CI, 94.0–96.0] (Fig. 5). The superiority in triage efficacy was most notable for the single-hs-cTnT model upon ED presentation triaging 32.1% versus 14.9% of patients. When the serial-hs-cTn model was applied, triage efficacy showed a modest improvement, triaging 77.1% versus 72.8% of patients.

#### Sensitivity analysis

The model without time since CPO showed comparable performance to the model with time since CPO (Supplemental Results, Supplemental Table S10 and Supplemental Figures S15 and S16).

## Discussion

In this large, prospective international multicentre diagnostic study of patients presenting to the ED with symptoms suggestive of AMI, we developed, internally and externally validated two ML-based models for a diagnosis of NSTEMI: i) a single-hs-cTn model using only data available at ED presentation and ii) a serial-hs-cTn model incorporating the second hs-cTn measurement and the time interval between measurements. We report seven major findings.

**First**, both models showed very high discrimination for NSTEMI in the derivation, internal validation, and external validation cohorts. **Second**, subgroup analysis showed lower performance in patients aged 65 and older, those with early-onset chest pain using the single hs-cTnT model, and those with kidney disease using the serial hs-cTnT model. **Third**, compared to the current guideline-recommended standard-of-care, the ESC 0/1 h-algorithm, FAST-NSTEMI triaged more patients towards rule-out and/or rule-in of NSTEMI in the internal and external validation cohorts with comparable safety and accuracy. The advantage in triage efficacy was most notable for the single-hs-cTnT model at ED presentation: 52.4% versus 29.8% of patients by the ESC 0/1 h-algorithm in the internal validation cohort, and 32.1% versus 14.9% in the external validation cohort. The lower triage efficacy in the external validation cohort was partially attributable to 26% missing CPO data, highlighting the need for simplified ML-based models that do not rely on the time since CPO. **Fourth**, although the FAST-NSTEMI model including CPO achieved higher safety in a biologically plausible manner given troponin kinetics, the simplified FAST-NSTEMI model excluding CPO still demonstrated excellent diagnostic performance. **Fifth**, sensitivity analysis with the second central adjudication using sex-specific rather than uniform URLs as the reference confirmed the excellent performance of FAST-NSTEMI in the early diagnosis of NSTEMI, enhancing its generalisability to centres using sex-specific URLs for hs-cTnT. **Sixth**, sensitivity analysis using hs-cTnI Architect instead of hs-cTnT showed the excellent performance of FAST-NSTEMI in the internal validation set. **Seventh**, SHAP explainability analysis showed that 0 h hs-cTnT concentration had the strongest impact on the single-hs-cTnT model, while the first serial hs-cTnT concentration had the strongest impact in the serial-hs-cTnT model.

These findings extend and corroborate three prior pioneering studies on ML-based diagnostic aids for patients presenting with suspected NSTEMI.^6^, ^7^, ^8^, ^9^, ^10^, ^11^ The myocardial-ischaemic-injury-index (MI^3^) algorithm uses hs-cTnI-Architect and computes a probability for type 1 NSTEMI based on age, sex, two serial hs-cTnI concentrations, and the time interval between them.^7^^,^^9^ It showed very high discrimination for NSTEMI type I, and overall comparable diagnostic performance to the ESC 0/1 h-algorithm. As it always requires two hs-cTnI measurements for computing an individualised probability, it cannot triage patients upon ED presentation with only the 0 h hs-cTn available, inevitably leading to a delay in decision making.^7^^,^^9^ Collaboration for the Diagnosis and Evaluation of Acute Coronary Syndrome (CoDE-ACS) also uses hs-cTnI-Architect and computes a probability for type 1 NSTEMI based on hs-cTnI and 12 additional variables.^7^ It was the first ML model to incorporate time from CPO. It showed very high discrimination for NSTEMI type I, and overall at least comparable diagnostic performance to the ESC 0/1 h-algorithm.^7^ Its limitations include a slightly lower sensitivity (<98%) and thereby safety for the selected threshold for the rule-out of NSTEMI in the external validation cohort.^13^ The Artificial intelligence in suspected myocardial infarction study (ARTEMIS) is an ML-based algorithm using different hs-cTnT/I assays and computes a probability for NSTEMI (type I + II) using 9 variables for the single hs-cTnT/I model and 8 variables for the serial hs-cTnT/I model. It showed very high discrimination for NSTEMI and overall comparable diagnostic performance to the ESC 0/1 h-algorithm. Its limitations include a rather small single-centre cohort for model derivation (n = 2575); thus, it is likely suffering from overfitting and lower generalisability, and consequently less robustness against performance drop in external validation. MI,^3^ CoDE-ACS, and ARTEMIS all remain proprietary, which restricts independent academic evaluation and clinical application.

FAST-NSTEMI at least in part addresses these limitations by using fewer, more objective variables, being less prone to recall bias, and developing the framework in two large international chest pain cohorts. The objective nature and low number of variables used in FAST-NSTEMI is one important strength and an important difference with respect to previous ML-based algorithms, which allow direct rule-out upon the first measured cTn (ARTEMIS and CoDE-ACS). As the number and complexity of required variables may pose significant implementation barriers, FAST-NSTEMI may be easier and safer to implement compared to previous models. Another shared strength of these ML tools is their ability to accommodate serial hs-cTn concentrations at flexible timepoints, potentially enhancing their applicability and feasibility compared to algorithms requiring a fixed timing for the second hs-cTn measurement, as e.g. the ESC 0/1 h- or ESC 0/2 h-algorithm. Whether this translates into shorter ED stays or faster, more accurate clinical decision-making remains to be shown.

Compared to the ESC 0/1 h-algorithm, FAST-NSTEMI maintained high triage effectiveness while increasing sensitivity and NPV. Importantly, FAST-NSTEMI output, similar to that of the other three ML-based models, is a continuous probability ranging from 0 to 100, thus allowing increased flexibility compared to current guideline-recommended algorithms, which are based on binary thresholds. For example, in a more conservative health care setting, the rule-out threshold for the single or serial-hs-cTnT model could be lowered, resulting in an even higher sensitivity and NPV for the rule-out of NSTEMI. Similarly, in health care settings with more restricted resources and high ED crowding, the use of higher rule-out thresholds could be justified to further increase triage efficacy and thereby decrease the length of stay in the ED. Accordingly, FAST-NSTEMI allows users to fine-tune the triage thresholds tailored to optimise safety, accuracy or efficacy based on local circumstances. However, the significant intercept offsets observed in external validation, primarily impacting the accuracy of rule-in decisions, suggest that local recalibration may be necessary before implementation to ensure portability across different settings.

The implications of deploying a ML-based decision support in acute care warrant careful consideration. FAST-NSTEMI outputs a continuous probability whose internal logic is not entirely transparent to the ED physician. This highlights the risk that an ML algorithm is followed in place of, rather than alongside, clinical judgement. FAST-NSTEMI is intended to support clinical assessment: the treating clinician retains responsibility for integrating the algorithm's output with patient's history, symptoms, vital signs, and the 12-lead ECG.

Several limitations merit consideration when interpreting these findings. First, APACE, TRAPID-AMI and RAPID-CPU were conducted in patients presenting to the ED. Therefore, we cannot comment on the performance of FAST-NSTEMI in other clinical settings. Second, although we used a very stringent methodology to adjudicate NSTEMI, including central adjudication by two independent cardiologists according to current guidelines and the 4th UDMI using cardiac imaging and serial measurements of local hs-cTn and hs-cTnT from study blood, we may still have misclassified a small number of patients. Third, hs-cTnT as a variable was part of the model and also, according to the 4th UDMI, part of the information available for central adjudication. Therefore, its weight in the model might have been inflated due to incorporation bias. Fourth, as a diagnostic study using centrally adjudicated NSTEMI as the reference standard, we cannot quantify implementation barriers and/or the effect of FAST-NSTEMI on medical and economic outcomes. Notably, the external validation relied on a single specialist tertiary centre with a dedicated chest pain unit and established accelerated diagnostic pathways (mainly the ESC 0/1 h-and 0/3 h-algorithms). Although 5220 patients were recruited, only 1915 were eligible for the comparison with the ESC 0/1 h-algorithm (227 NSTEMI events), a modest sample for confirming safety in the low-risk rule-out group. Generalisability of the models' performance and the derived triage thresholds in community or non-specialist EDs, and in non-European populations remains to be shown. The clinical utility of FAST-NSTEMI as a decision-support tool should ideally be evaluated in large prospective implementation studies. Fifth, FAST-NSTEMI has been trained and validated using the currently best-validated and most widely used hs-cTnT/I-assay, ie, hs-cTnT-gen5.^18^ Despite demonstrating good diagnostic performance in internal validation with the hs-cTnI-Architect assay, external validation in cohorts using the hs-cTnI-Architect or other hs-cTn assay is needed to further confirm the generalisability of the model. Sixth, as competing ML tools (MI^3^, CoDE-ACS, and ARTEMIS) remain proprietary, independent direct performance comparisons were not feasible.^7^^,^^9^ However, as our model is open-access, this direct comparison is now possible and strongly encouraged for those groups having this proprietary information. Seventh, the ECG criteria were established through cardiologist interpretation and one of the four criteria, namely T wave inversion, showed only limited impact. Ongoing efforts to integrate AI-based ECG analysis with hs-cTn and clinical variables may further enhance efficiency, consistency, and overall model performance. Eighth, the inclusion of type 2 NSTEMI might obscure the model's true accuracy for the pathophysiologically distinct type 1 cohort, where rapid triage is most critical.

In conclusion, FAST-NSTEMI is an open-access ML-based decision support tool with very high discrimination and good calibration for NSTEMI using single and serial-hs-cTnT models. Compared to the ESC hs-cTnT-0/1 h-algorithm, it safely triages a greater proportion of patients at presentation. As a scalable and transparent tool, FAST-NSTEMI could reduce ED length of stay and improve care efficiency across diverse healthcare settings.

## Contributors

AC, PL-A, IS, and CM conceived the study and its design. IS and CM acted as supervisors for the study. CR performed the external validation under the supervision of EG, who is responsible for the data collection in the external centre. All authors, including PC, LC, KD, JSS, FM and BL, were involved in data collection and curation. OM, FJM-S, DIK, BM, MC, JP, and CM provided resources for the study. AC, PL-A, JB, TZ, LK, EK, PB, LH-R, GH and CM had access to and verified the raw data. AC and PL-A performed the data analysis. IS validated the analysis. AC, PL-A, JB, KW, IS, and CM interpreted the data. AC, PL-A, IS, and CM draughted the manuscript. DH developed and deployed the online applications. All authors had full access to the data presented in the study. All authors revised and edited the manuscript critically for important intellectual content. All authors provided their final approval of the version to be published. All authors accept responsibility to submit for publication. APACE and TRAPID-AMI investigators contributed to data generation.

## Data sharing statement

The analysis code that supports the findings of this study and the raw algorithm are available online: https://github.com/arnaudMLchamp/XGBoost-for-NSTEMI-detection-using-TnT.

The data to confirm the results can be shared upon reasonable request. Two online applications allowing beta testing of the proposed models are publicly accessible. Version 1.0 incorporates time since onset of chest pain, while version 1.1 does not include this variable. The applications are available at the following links: https://arnaudmlchamp.shinyapps.io/fast-nstemi-v10/ and https://arnaudmlchamp.shinyapps.io/fast-nstemi-v11/.

## Declaration of interests

PLA has received research grants from the Swiss Heart Foundation (FF20079, FF21103, and FF24149) and speaker honoraria from Quidel, Roche Diagnostics, and Polymedco, all outside the submitted work. CR reports institutional research support from the University of Heidelberg (Clinician Scientist Stipendium), grants from AstraZeneca and the University of Heidelberg, speaker honoraria from AstraZeneca, Roche, and Thermofisher, travel grants from DZHK, AstraZeneca, and Bayer, a patent pending at University Hospital Heidelberg, and participation in an AstraZeneca Advisory Board, all outside the submitted work. CM reports research grants/support from the Swiss National Science Foundation, the University of Basel, the University Hospital Basel, the Swiss Heart Foundation, Innosuisse, Abbott, Astra Zeneca, Beckman Coulter, Brahms, Idorsia, LSI Medience Corporation, Mindray, Novartis, Ortho Diagnostics, Quidel, Roche, Siemens, SpinChip, Singulex, and Sphingotec; all paid to the institution; consulting fees from Abbott, Brahms, Idorsia, Roche, and SpinChip; and honoraria for lectures and support for attending meetings/travel from Abbott, Amgen, AstraZeneca, Bayer, Boehringer Ingelheim, BMS, Idorsia, Novartis, Roche, and Sanofi, all outside the submitted work. FM reports grants or contracts from the Deutsche Forschungsgemeinschaft (SFB TRR219), the Deutsche Gesellschaft für Kardiologie (DGK), the Deutsche Herzstiftung, Ablative Solutions, and ReCor Medical; consulting fees, payment or honoraria for lectures, and support for attending meetings/travel from Ablative Solutions, AstraZeneca, Novartis, Inari, ReCor Medical, Medtronic, Philips, and Merck; and a leadership role as a Board member of the European Society of Cardiology, all outside the submitted work. EG reports consulting fees from Roche Diagnostics and Brahms Deutschland, and payment or honoraria for lectures from AstraZeneca, Roche Diagnostics, Bayer Vital, Amgen, Brahms Deutschland, Novartis, and Daiichi Sankyo (all paid to him), outside the submitted work. OM reports consulting fees from BioMerieux and payment or honoraria for lectures from Gilead, outside the submitted work. JB reports payment or honoraria for lectures from Beckman Coulter and Teleflex, and support for attending meetings/travel from VascularMedical, outside the submitted work. LK reports speaker honoraria from Roche, Abbott, Dr. Risch, and Siemens, and travel expenses covered by Roche, outside the submitted work. EK reports grants from the Swiss Heart Foundation, University Hospital Basel, the Bangerter-Rhyner Foundation, and the Freiwillige Akademische Gesellschaft Basel, consulting fees from SpinChip, and payment or honoraria for lectures from Medtrix AG Basel, outside the submitted work. KW reports grants from the Swiss National Science Foundation, University of Basel, and Wesley Medical Research Institute (Brisbane, Australia), consulting fees from SpinChip, and payment or honoraria from the University of Zurich, all unrelated to this work. PB reports consulting fees from Aurevia (paid to him), outside the submitted work. BL reports a grant from the Swedish Heart and Lung Foundation. KD has received research grants from the Swiss Heart Foundation (FF25099). All other authors declare no competing interests relevant to this publication.
