# Supplementary material for: Machine learning for diagnosing non-ST-segment elevation myocardial infarction: a derivation and validation study
Source: eClinicalMedicine. 2026 Jul 9;97:104055. doi: 10.1016/j.eclinm.2026.104055 (PMC13380782; doi:10.1016/j.eclinm.2026.104055)
Supplement: Supplementary Materials [file mmc1.docx]

SUPPLEMENTAL MATERIAL

**Machine learning for diagnosis of non-ST-segment myocardial infarction: a derivation and validation study**

**Supplemental Methods**

**Blood sampling and laboratory methods**

**External validation: Rapid-CPU**

**The European Society of Cardiology 0/1h-algorithm**

**The XGBoost algorithm**

**List of Python libraries used for the model derivation**

**Model derivation and internal validation**

**Distributions grid used to sample hyperparameters**

**Statistical analysis**

**List of R packages used for the analysis**

**Explainability**

**Supplemental Results**

**Internal validation**

**Triage thresholds**

**Sensitivity analysis 1: model without time since CPO**

**SHAP**

**External validation**

**Triage thresholds**

**Sensitivity analysis: Model without CPO**

**Supplemental Tables**

**Supplemental Table 1** –Baseline characteristics of the upsampled cohorts for derivation and internal validation of FAST-NSTEMI with single and serial hs-cTnT measurements

**Supplemental Table 2** – Baseline Characteristics of the APACE cohort per centre

**Supplemental Table 3** – Baseline Characteristics of the TRAPID-AMI cohort per centre

**Supplemental Table 4** – Diagnostic thresholds of FAST-NSTEMI in the derivation cohorts

**Supplemental Table 5** – Diagnostic thresholds of FAST-NSTEMI without time since onset of chest pain in the derivation cohorts

**Supplemental Table 6** – Diagnostic thresholds of FAST-NSTEMI without time since onset of chest pain in the internal validation cohorts

**Supplemental Table 7** – Diagnostic thresholds of FAST-NSTEMI using the central adjudication based on sex specific URLs as the reference in the internal validation cohort

**Supplemental Table 8** – Diagnostic thresholds of FAST-NSTEMI in the external validation cohorts

**Supplemental Table 9** – Diagnostic thresholds of FAST-NSTEMI in the external validation cohorts with liberal imputation of the time since onset of chest pain

**Supplemental Table 10** – Diagnostic thresholds of FAST-NSTEMI without time since the onset of chest pain in the external validation cohorts

**Supplemental Table 11** – Baseline characteristics of the APACE and TRAPID-AMI cohort per sex

**Supplemental Figure Legends**

**Supplemental Figure 1** **– Patient flowchart of the APACE and TRAPID-AMI pooled cohort.**

STEMI = ST-segment elevation myocardial infarction; URL = upper reference limit; ECG = Electrocardiogram; hs-cTnT = high-sensitivity cardiac troponin T

**Supplemental Figure 2** **–**  **Patient flowchart including the up-sampling of the cohort and its split for the derivation and internal validation of the single hs-cTnT model.**

STEMI = ST-segment elevation myocardial infarction; URL = upper reference limit; ECG = Electrocardiogram; hs-cTnT = high-sensitivity cardiac troponin T

**Supplemental Figure 3** **–** **Patient flowchart including the up-sampling of the cohort and its split for the derivation and internal validation of the serial hs-cTnT** **model.**

STEMI = ST-segment elevation myocardial infarction; URL = upper reference limit; ECG = Electrocardiogram; hs-cTnT = high-sensitivity cardiac troponin T

**Supplemental Figure 4** **–** **Patient flowchart of the RAPID-CPU cohort.**

STEMI = ST-segment elevation myocardial infarction; URL = upper reference limit; ECG = Electrocardiogram; hs-cTnT = high-sensitivity cardiac troponin T

**Supplemental Figure 5. Diagnostic performance of the FAST-NSTEMI in the upsampled versus non-upsampled derivation cohort.**

(A & E) Receiver-operating-characteristic curve showing the discrimination of FAST-NSTEMI for NSTEMI using a single hs-cTnT measurement in, (A) the upsampled and, (E) the non-upsampled cohorts; (B & F) Calibration curve of the FAST-NSTEMI algorithm using a single hs-cTnT measurement in, (B) the upsampled and, (F) the non-upsampled cohorts, assessing the agreement between predicted probabilities and observed proportion. The red line represents optimal calibration, the black solid line represents the calibration curve derived using loess smoothing while the green line represents the calibration curve derived using restricted cubic splines, and the black dashed line represents the 95% confidence interval of the calibration curve.

(C & G) Receiver-operating-characteristic curve showing the discrimination of FAST-NSTEMI for NSTEMI using two consecutive hs-cTnT measurements in, (C) the upsampled and, (G) the non-upsampled cohorts; (D & H) Calibration curve of the FAST-NSTEMI algorithm using two consecutive hs-cTnT measurements, assessing the agreement between predicted probabilities and observed proportion in, (D) the upsampled and, (H) the non-upsampled cohorts.

hs-cTnT = high-sensitivity cardiac troponin T; AUC = Area under the receiver operating characteristic curve.

**Supplemental Figure 6. AUC of the FAST-NSTEMI across patient subgroups.**

(A) Single hs-cTnT model predictions.

(B) Serial hs-cTnT model predictions.

hs-cTnT = high-sensitivity cardiac troponin T; AUC = area under the receiver operating characteristic curve

**Supplemental Figure 7** **–** **Proportion of ruled-in and ruled-out patients at probabilistic thresholds and their associated performance.**

(A) Proportion of ruled-out patients and associated NPV and sensitivity at different probability thresholds, as calculated by the FAST-NSTEMI model using a single hs-cTnT measurement; (B) Proportion of ruled-in patients and associated PPV and specificity at different probability thresholds, as calculated by the FAST-NSTEMI model using a single hs-cTnT measurement; (C) Proportion of ruled-out patients and associated NPV and sensitivity at different probability thresholds, as calculated by the FAST-NSTEMI model using two consecutive hs-cTnT measurements; (D) Proportion of ruled-in patients and associated PPV and specificity at different probability thresholds, as calculated by the FAST-NSTEMI model using two consecutive hs-cTnT measurements; NPV = negative predictive value; PPV = positive predictive value. hs-cTnT = high-sensitivity cardiac troponin T.

**Supplemental Figure 8. Diagnostic performance of the FAST-NSTEMI versus the recalibrated TIMI and HEART scores.**

hs-cTnT = high-sensitivity cardiac troponin T; AUC = area under the receiver operating characteristic curve; NSTEMI = non-ST-segment elevation myocardial infarction

**Supplemental Figure 9. Diagnostic performance of the FAST-NSTEMI when used with high-sensitive cardiac troponin I Architect.**

(A) Distribution of predicted probabilities as calculated by FAST-NSTEMI using a single hs-cTnI Architect measurement; (B) Receiver-operating-characteristic curve showing the discrimination of FAST-NSTEMI for NSTEMI using a single hs-cTnI Architect measurement; (C) Calibration curve of the FAST-NSTEMI algorithm using a single hs-cTnI Architect measurement, assessing the agreement between predicted probabilities and observed proportion. The red line represents optimal calibration, the black solid line represents the calibration curve derived using loess smoothing while the green line represents the calibration curve derived using restricted cubic splines, and the black dashed line represents the 95% confidence interval of the calibration curve.

(D) Distribution of predicted probabilities as calculated by FAST-NSTEMI using two consecutive hs-cTnI Architect measurements; (E) Receiver-operating-characteristic curve showing the discrimination of FAST-NSTEMI for NSTEMI using two consecutive hs-cTnI Architect measurements; (F) Calibration curve of the FAST-NSTEMI algorithm using two consecutive hs-cTnI Architect measurements, assessing the agreement between predicted probabilities and observed proportion.

hs-cTnI = high-sensitivity-cardiac troponin I ; AUC = Area under the receiver operating characteristic curve.

**Supplemental Figure 10. Diagnostic performance of the FAST-NSTEMI without time since onset of chest pain in the internal validation cohort.**

(A) Distribution of predicted probabilities as calculated by FAST-NSTEMI using a single hs-cTnT measurement; (B) Receiver-operating-characteristic curve showing the discrimination of FAST-NSTEMI for NSTEMI using a single hs-cTnT measurement; (C) Calibration curve of the FAST-NSTEMI algorithm using a single hs-cTnT measurement, assessing the agreement between predicted probabilities and observed proportion. The red line represents optimal calibration, the black solid line represents the calibration curve derived using loess smoothing while the green line represents the calibration curve derived using restricted cubic splines, and the black dashed line represents the 95% confidence interval of the calibration curve.

(D) Distribution of predicted probabilities as calculated by FAST-NSTEMI using two consecutive hs-cTnT measurements; (E) Receiver-operating-characteristic curve showing the discrimination of FAST-NSTEMI for NSTEMI using two consecutive hs-cTnT measurements; (F) Calibration curve of the FAST-NSTEMI algorithm using two consecutive hs-cTnT measurements, assessing the agreement between predicted probabilities and observed proportion.

hs-cTnT = high-sensitivity cardiac troponin T; AUC = Area under the receiver operating characteristic curve.

**Supplemental Figure 11. Thresholds performance of the FAST-NSTEMI without time since onset of chest pain in the internal validation cohort.**

ESC = European Society of Cardiology; NSTEMI = non-ST-segment elevation myocardial infarction; NPV = negative predictive value; PPV = positive predictive value; FN = false negative; CPO = chest pain onset; hs-cTnT = high-sensitivity cardiac troponin T.

**Supplemental Figure 12. Diagnostic performance of the FAST-NSTEMI using sex specific cut-off gold standard diagnosis in the internal validation cohort.**

(A) Distribution of predicted probabilities as calculated by FAST-NSTEMI using a single hs-cTnT measurement; (B) Receiver-operating-characteristic curve showing the discrimination of FAST-NSTEMI for NSTEMI using a single hs-cTnT measurement; (C) Calibration curve of the FAST-NSTEMI algorithm using a single hs-cTnT measurement, assessing the agreement between predicted probabilities and observed proportion. The red line represents optimal calibration, the black solid line represents the calibration curve derived using loess smoothing while the green line represents the calibration curve derived using restricted cubic splines, and the black dashed line represents the 95% confidence interval of the calibration curve.

(D) Distribution of predicted probabilities as calculated by FAST-NSTEMI using two consecutive hs-cTnT measurements; (E) Receiver-operating-characteristic curve showing the discrimination of FAST-NSTEMI for NSTEMI using two consecutive hs-cTnT measurements; (F) Calibration curve of the FAST-NSTEMI algorithm using two consecutive hs-cTnT measurements, assessing the agreement between predicted probabilities and observed proportion.

hs-cTnT = high-sensitivity cardiac troponin T; AUC = Area under the receiver operating characteristic curve.

**Supplemental Figure 13. Diagnostic performance of the FAST-NSTEMI using 0/2h and 0/3h high-sensitivity cardiac troponin T values in the internal validation cohort.**

Distribution of predicted probabilities as calculated by FAST-NSTEMI using 0 and 2h hs-cTnT measurements; (B) Receiver-operating-characteristic curve showing the discrimination of FAST-NSTEMI for NSTEMI using 0 and 2h hs-cTnT measurements; (C) Calibration curve of the FAST-NSTEMI algorithm using 0 and 2h hs-cTnT measurements, assessing the agreement between predicted probabilities and observed proportion.

(D) Distribution of predicted probabilities as calculated by FAST-NSTEMI using 0 and 3h hs-cTnT measurements; (E) Receiver-operating-characteristic curve showing the discrimination of FAST-NSTEMI for NSTEMI using 0 and 3h hs-cTnT measurements; (F) Calibration curve of the FAST-NSTEMI algorithm using 0 and 3h hs-cTnT measurements, assessing the agreement between predicted probabilities and observed proportion.

hs-cTnT = high-sensitivity cardiac troponin T; AUC = Area under the receiver operating characteristic curve.

**Supplemental Figure 14. Proportion of ruled-in and ruled-out patients at probabilistic thresholds and their associated performance in the external validation cohort.**

(A) Proportion of ruled-out patients and associated NPV and sensitivity at different probability thresholds, as calculated by the FAST-NSTEMI model using a single hs-cTnT measurement; (B) Proportion of ruled-in patients and associated PPV and specificity at different probability thresholds, as calculated by the FAST-NSTEMI model using a single hs-cTnT measurement; (C) Proportion of ruled-out patients and associated NPV and sensitivity at different probability thresholds, as calculated by the FAST-NSTEMI model using two consecutive hs-cTnT measurements; (D) Proportion of ruled-in patients and associated PPV and specificity at different probability thresholds, as calculated by the FAST-NSTEMI model using two consecutive hs-cTnT measurements; NPV = negative predictive value; PPV = positive predictive value. hs-cTnT = high-sensitivity cardiac troponin T

**Supplemental Figure 15. Diagnostic performance of the FAST-NSTEMI without time since onset of chest pain in the external validation cohort.**

(A) Receiver-operating-characteristic curve showing the discrimination of FAST-NSTEMI for NSTEMI using a single hs-cTnT measurement; (B) Calibration curve of the FAST-NSTEMI algorithm using a single hs-cTnT measurement, assessing the agreement between predicted probabilities and observed proportion. The red line represents optimal calibration, the black solid line the calibration curve derived using loess smoothing while the green line represents the calibration curve derived using restricted cubic splines, and the black dashed line the 95% confidence interval of the calibration curve. (C) Calibration curve of the FAST-NSTEMI algorithm using a single hs-cTnT measurement, assessing the agreement between intercept-corrected predicted probabilities and observed proportion.

(D) Receiver-operating-characteristic curve showing the discrimination of FAST-NSTEMI for NSTEMI using two serial hs-cTnT measurements; (E) Calibration curve of the FAST-NSTEMI algorithm using two serial hs-cTnT measurements, assessing the agreement between predicted probabilities and observed proportion. (F) Calibration curve of the FAST-NSTEMI algorithm using two serial hs-cTnT measurements, assessing the agreement between intercept-corrected probabilities and observed proportion.

Hs-cTnT = High-sensitive cardiac troponin T; AUC = Area under the receiver operating characteristic curve

**Supplemental Figure 16. Performance of the FAST-NSTEMI without time since onset of chest pain in the external validation cohort.**

ESC = European Society of Cardiology; NSTEMI = non-ST-segment elevation myocardial infarction; NPV = negative predictive value; PPV = positive predictive value; FN = false negative; CPO = chest pain onset; hs-cTnT = high-sensitivity cardiac troponin T.

**Supplemental Figure 17. Theoretical trees to illustrate SHAPley values computations.**

(A) Tree with asymmetric creatinine effects.

(B) Tree with symmetric creatinine effects.

**Supplemental Figure 18. Force plots of a patient using FAST-NSTEMI.**

(A) Effect of each variable on a single patient prediction using the single hs-cTnT model as computed by SHAP.

(B) Effect of each variable on a single patient prediction using the serial hs-cTnT model as computed by SHAP.

**Supplemental Figure 19. Heat maps of probabilities of being ruled out, ruled in, or kept in the observed zone using the single hs-cTnT** **model.**

(A) Probabilities of being ruled out or kept in the observe-zone for patients with time since chest pain onset of 54 min, varying 0h hs-cTnT and age, and median values for other variables.

(B) Probabilities of being ruled out or kept in the observe-zone for patients with time since chest pain onset of 1 hour 30 min, varying 0h hs-cTnT and age, and median values for other variables.

(C) Probabilities of being ruled in or kept in the observe-zone for patients with time since chest pain onset of 6 hours, varying 0h hs-cTnT and age, and median values for other variables.

(D) Probabilities of being ruled in or kept in the observe-zone for patients with time since chest pain onset of 15 hours, varying 0h hs-cTnT and age, and median values for other variables.
Median values are: creatinine=87umol/L; sex=male; Normal ECG.

CPO=chest pain onset

**Supplemental Methods**

**Blood sampling and laboratory methods**

Blood samples for determination of high-sensitivity cardiac troponin (hs-cTn)T-gen5 (Roche Diagnostics, Penzberg, Germany) were collected into heparin plasma tubes at ED presentation and serially thereafter. Plasma creatinine concentration was measured as part of the local standard of care. Serial sampling was discontinued when a patient was discharged or transferred to the catheterisation laboratory for treatment. After centrifugation, samples were frozen at -80°C until assayed in a blinded fashion in a dedicated core laboratory. The Roche hs-cTnT assays used the Elecsys 2010 system (Roche Diagnostics, Rotkreuz, Switzerland), with a limit of detection of 3-5ng/L depending on the analyser used, a 99th-percentile upper-reference limit (URL) of 14ng/L, and a coefficient of variation of less than 10% at 13ng/L. The estimated glomerular filtration rate was determined using the chronic kidney disease epidemiology collaboration formula. The hs-cTnI Architect assay (ARCHITECT STAT hs-cTnI, Abbott Laboratories) has a 99th percentile upper reference limit of 26 ng/L (women, 16 ng/L; men, 34 ng/L) with a corresponding CV of <5% and a limit of detection of 1.9 ng/L. ^11^

**External validation: Rapid-CPU**

Patient management occurred within a specialised chest pain unit (CPU), a cardiologist-led section of the ED certified by the German Cardiac Society. Precise electronic time stamps were available to document key events, including arrival, referral, discharge, blood sample collection (and intervals between samples), reporting of test results ("turnaround times"), and all diagnostic and therapeutic procedures. The cohort was subjected to the same exclusion criteria as those used in deriving FAST-NSTEMI. Medication use at the time of presentation was not recorded.

Hs-cTnT levels were measured upon arrival and again one hour later (±30 minutes) using the Cobas E 411 system (Roche Diagnostics Ltd., Rotkreuz, Switzerland). Two independent cardiologists made a final adjudicated diagnosis in both populations—a third cardiologist referred in cases of disagreement. The adjudication was based on all available clinical and imaging parameters (e.g., ECG, Echo, MRI, Angiography) and hs-cTnT. The criteria for the diagnosis of MI were based on the fourth universal MI definition.^2^

In contrast to APACE and TRAPID-AMI, time since CPO was not recorded as a continuous variable, but as a categorical variable as follows: 0–3 hours, 3–6 hours, >6 hours, and unknown. Since the proportion of missing values for time since CPO was 26%, it was decided to impute it. For conservative decision-making, the following assumptions were applied: patients with time since CPO of 0–3 hours or unknown were assigned a value of <1 hours (i.e. inserted as 09·), reflecting the early onset assumption that patients would need a second hs-cTnT measurement. Patients in the 3–6-hour category were assigned a value of 4.5 hours, corresponding to the midpoint of this interval. Patients in the >6-hour category were assigned a value of 6 hours, representing the earliest possible onset within this group.

To further evaluate the performance of the model, a sensitivity analysis with modified imputation of time since CPO was conducted. Based on observations from the model derivation process, alternative time assumptions were applied for specific groups. Patients with time since CPO of 0–3 hours were assigned a value of 1.5 hours **(Supplemental Figure 19 B)**, representing a slightly later onset within this category. Similarly, patients in the >6-hour group were assigned a value of 15 hours, reflecting a later onset assumption for this category **(Supplemental Figure 19 D)**.

**The European Society of Cardiology 0/1h-algorithm**

The European Society of Cardiology **(**ESC) hs-cTnT-0/1h-algorithm was applied as recommended in current guidelines.^3,4^ In the internal validation dataset, the comparison between FAST-NSTEMI and the ESC hs-cTnT-0/1h-algorithm was limited to patients with a maximum interval of 90 minutes between consecutive blood draws. The ESC 0/1h algorithm is independent of chest pain characteristics or clinical scores, using only assay-specific hs-cTn cut-offs for rapid triage of patients with symptoms suggestive of AMI. The ESC 0/1h-hs-cTnT algorithm considers values of hs-cTnT drawn at the ED presentation and again after 1h±20min. Very low hs-cTnT concentrations at presentation allow for an early rule out of AMI and often also early discharge of patients without requiring a second blood draw if the time from chest pain onset to presentation is >3h. Very high concentrations of hs-cTnT at presentation or a relevant 0/1h absolute change allow for an early rule in AMI. The ESC 0/1h-hs-cTnT algorithm was derived and validated for index type 1 or type 2 NSTEMI.

**The XGBoost algorithm**

In XGBoost, predictions are built up iteratively by adding new trees that correct the errors of previous predictions:

$${\hat{\boldsymbol{y}}}_{\boldsymbol{i}}^{\left( \boldsymbol{t} \right)}\boldsymbol{=}{\hat{\boldsymbol{y}}}_{\boldsymbol{i}}^{\left( \boldsymbol{t-1} \right)}\boldsymbol{+}\boldsymbol{f}_{\boldsymbol{t}}\left( \boldsymbol{x}_{\boldsymbol{i}} \right)$$

Where:

- ${\hat{\boldsymbol{y}}}_{\boldsymbol{i}}^{\left( \boldsymbol{t} \right)}$: the prediction for sample i at iteration t.
- ${\hat{\boldsymbol{y}}}_{\boldsymbol{i}}^{\left( \boldsymbol{t-1} \right)}$: the prediction for sample i of the prior iteration, t-1.
- $\boldsymbol{f}_{\boldsymbol{t}}\left( \boldsymbol{x}_{\boldsymbol{i}} \right)$: the new tree at iteration t that provides an update

The objective function in XGBoost combines a loss function to measure prediction accuracy and a regularisation term to control model complexity:

$$\mathcal{L}^{\boldsymbol{(t)}}=\sum_{i=1}^{\boldsymbol{n}} \boldsymbol{l}\left( \boldsymbol{y}_{\boldsymbol{i}}\boldsymbol{,}{\hat{\boldsymbol{y}}}_{\boldsymbol{i}}^{\left( \boldsymbol{t-1} \right)}\boldsymbol{+}\boldsymbol{f}_{\boldsymbol{t}}\left( \boldsymbol{x}_{\boldsymbol{i}} \right) \right)+\boldsymbol{\Omega}\left( \boldsymbol{f}_{\boldsymbol{k}} \right)$$

where:

- $\mathcal{L}^{\boldsymbol{(t)}}$ is the total objective function we want to minimize, combining loss and regularization at iteration t.
- $n$ is the number of training examples.
- $\boldsymbol{l}\left( \boldsymbol{y}_{\boldsymbol{i}}\boldsymbol{,}{\hat{\boldsymbol{y}}}_{\boldsymbol{i}}^{\left( \boldsymbol{t} \right)} \right)$ is the loss function, we opted for the log-likelihood defined as:

$$\boldsymbol{l}\left( \boldsymbol{y}_{\boldsymbol{i}}\boldsymbol{,}{\hat{\boldsymbol{y}}}_{\boldsymbol{i}}^{\left( \boldsymbol{t} \right)} \right)\boldsymbol{=-}\boldsymbol{y}_{\boldsymbol{i}}\mathbf{lo}\mathbf{g} {\hat{\boldsymbol{y}}}_{\boldsymbol{i}}^{\left( \boldsymbol{t} \right)}\boldsymbol{-}\left( \boldsymbol{1-}\boldsymbol{y}_{\boldsymbol{i}} \right)\mathbf{lo}\mathbf{g} \left( \boldsymbol{1-}{\hat{\boldsymbol{y}}}_{\boldsymbol{i}}^{\left( \boldsymbol{t} \right)} \right)$$

Where $\boldsymbol{y}_{\boldsymbol{i}}$ is the true label, and ${\hat{\boldsymbol{y}}}_{\boldsymbol{i}}^{\left( \boldsymbol{t} \right)}$ the model’s prediction at iteration t.

- $\boldsymbol{\Omega}\left( \boldsymbol{f}_{\boldsymbol{k}} \right)$ is the regularisation term for the k^th^ tree $\boldsymbol{f}_{\boldsymbol{k}}$, which penalises the complexity of the tree to prevent overfitting. This term is defined as:

$$\boldsymbol{\Omega}\left( \mathbf{f}_{\mathbf{k}} \right) = \boldsymbol{\gamma} \boldsymbol{T}_{\boldsymbol{k}} + \boldsymbol{\alpha} \sum_{j=1}^{T_{k}} \left| \boldsymbol{w}_{\boldsymbol{j}_{\boldsymbol{k}}} \right| + \frac{1}{2} \boldsymbol{\lambda} \sum_{j=1}^{T_{k}} \boldsymbol{w}_{\boldsymbol{j}_{\boldsymbol{k}}^{2}}$$

Where $\boldsymbol{w}_{\boldsymbol{j}}$ are the weights associated with each leaf j of the tree; $\boldsymbol{T}_{\boldsymbol{k}}$ represents the number of leaf nodes in the **k**^th^ tree; the hyperparameter $\boldsymbol{\gamma}$(gamma) penalises the number of leaf nodes in each tree; $\boldsymbol{\alpha}$ (alpha) is the weight for the L1 regularisation term and $\boldsymbol{\lambda}$ (lambda) is the weight for the L2 regularisation term.

**List of Python libraries used for the model derivation**

pandas; xgboost; shap; pickle; matplotlib; sklearn; scipy.

**Model derivation and internal validation**

In accordance with established methodological guidance for clinical prediction modeling, the FAST-NSTEMI model was derived using a pre-specified set of clinically plausible predictors; exclusion of CPO was informed by post hoc derivation considerations and motivated by practical implementation needs.^5^ Variables with smaller SHAP contributions were retained to preserve complementary predictive information, while model simplification was guided by clinical feasibility and data availability rather than post hoc importance metrics, and therefore applied specifically to chest pain onset. Hyperparameter tuning was conducted on the training set by sampling from predefined distributions. To minimise optimism, a nested cross-validation approach was employed. The Brier score served as the criterion for selecting the best-performing hyperparameter combination. The Brier score was selected because it integrates both discrimination and calibration metrics into a single measure.^5^ On average, the optimal combination was identified after 12,183 samplings (min: 3964, max: 27515). Due to computational and time constraints, the number of samplings was limited to 30,000. Other modelling strategies were explored, including logistic regression with restricted cubic splines to better capture non-linear effects of continuous variables, as well as an early version of TabPFN. However, the logistic regression model demonstrated lower discriminatory performance, and the selected decision thresholds did not yield better results than the XGBoost model. TabPFN produced miscalibrated probability estimates in the derivation cohort and was therefore not retained for further analyses.^6,7^ Other deep or machine learning methods were not considered due to their increased complexity and higher risk of overfitting. No clustering method were employed to account for intra-patient variability after the upsampling strategy. However, to prevent patient-level data leakage during cross-validation, a GroupKFold strategy was used ensuring that all measurements from a given patient were assigned to the same fold. The models were developed using Python.^8^ The model development methodology has not been previously described; however, the code for deriving the models, along with the models' weights, is available on GitHub (<https://github.com/arnaudMLchamp/XGBoost-for-NSTEMI-detetection-using-TnT>).

**Distributions used to sample hyperparameters**

{"max_depth": range(3, 11).

"alpha" : scipy.stats.uniform(loc = 0, scale = 20),

"gamma" : scipy.stats.uniform(loc = 1, scale = 20),

"lambda" : scipy.stats.uniform(loc = 0, scale = 20),

"colsample_bytree": scipy.stats.uniform(loc = 0, scale = 1),

"n_estimators": range(150, 1500),

"subsample": scipy.stats.uniform(loc = 0, scale = 1),

"min_child_weight": range(1, 15)}

A description of those parameters can be found here: https://xgboost.readthedocs.io/en/stable/parameter.html

**Statistical analysis**

The discrimination of FAST-NSTEMI was further evaluated in pre-specified subgroups: age (stratified by 65 years), sex, diabetes, history of coronary artery disease (CAD), early presenters (defined as time since CPO ≤ 3 hours), and renal failure (defined as an Estimated glomerular filtration rate [eGFR] < 60 mL/min/1.73 m2). Calibration intercept and calibration slope were also reported. To adjust for the different baseline risks in the external validation cohort, the models were recalibrated by refitting the intercept of a logistic regression model. This was achieved by incorporating the log of the predicted odds as a fixed offset term in the regression.

To address the issue of multiple comparisons for the different performance measures of the models/algorithms, we prioritised the comparison of the proportion of patients remaining in the observe zone. In the internal validation cohort, two sensitivity analyses were performed: one using as a reference the central adjudication with sex-specific URLs, and one using models without time since CPO.^15^

**List of R packages used for the statistical analysis**

dplyr; haven; caret; glue; readr; tidyr; ggbreak; CalibrationCurves; pROC; stringr; xgboost; Matrix.

**Explainability**

SHAP (SHapley Additive exPlanations) values quantify the contribution of each feature to a single model's prediction. In this example, two decision trees are used to explain how SHAP values are calculated. Each tree splits patients based on troponin and creatinine levels, with the leaf nodes showing scores corresponding to the log odds of the endpoint (**Supplemental Figure 17 A)**.

In both **Supplemental Figure 17 A and Supplemental Figure 17 B**, the baseline score represents the average score across all paths and is calculated as:

$$\frac{\left( -3*25 - 2.8*25 + 0.3*25 + 0.1*25 \right)}{4*25} = -1.35.$$

This baseline score value is shown in the first node. To calculate the SHAP values, we first determine the difference between each leaf score and the baseline score.

For example, in **Supplemental Figure 17 A**, for patients with troponin ≤ 8 and creatinine ≤ 87.5, the score is -3, yielding a difference of -1.65 from the baseline score. This difference captures the combined contribution of troponin and creatinine on the prediction. The contribution of creatinine among this group is -0.1 (-3-(-2.9)). These contributions are then averaged by the proportion of patients present in each of these splits (25/50). When troponin $\leq$ 8 the contribution of creatinine $\leq$87.5 is then -0.05. We can then compute the contribution of troponin for this group of patients by subtracting the contribution of creatinine from the initial difference from the baseline score: -1.65 - (-0.05) = -1.6. Therefore, any patient having both troponin $\leq$8 and creatinine $\leq$87.5 will have a SHAP value of troponin equal to -1.6 and creatinine equal to -0.05. Using the same methodology, we could compute the contribution of troponin (order from right to left in **Supplemental Figure 17 A**: -1.6, -1.5, 1.6, 1.5) and creatinine (-0.05, 0.05, 0.05, -0.05) among each group of patients.

In this scenario, the impact of the variable troponin on the magnitude of the outcome would be the average of the absolute value of its contribution across each leaf:

$$\frac{\left| -1.6 \right|+\left| -1.5 \right|+\left| 1.6 \right|+\left| 1.5 \right|}{4}=1.55$$

In **Supplemental Figure 17 B**, following the same method, we can see that the effect of creatinine is constant over the split (-0.1 if creatinine is $\leq$87.5 and +0.1 if creatinine is> 87.5). The proportion of patients experiencing these effects is 100%. Thus, among the patients with troponin $\leq$ 8 creatinine $\leq$ 87.5, the SHAP value of troponin < 8 is now -1.65 - (-0.1) = -1.55, and the SHAP value of creatinine < 87.5 is -0.1.

This example is simple and theoretical. However, to compute individual SHAP values, one must consider all trees built by the algorithm. The patients will “fall” into all the paths built by the iterative trees and will have a unique effect from each of the variables, computed in a similar way to how we did. The effect of the variables on a single prediction using models 1 and 2 is pictured on the corresponding force plots in **Supplemental Figures 18 A and B**. Each variable’s effect on the predictions of every patient from the validation dataset is depicted by a single point inside the summary plot in **Figures 1 A and B,** alongside the impact of each variable on the magnitude of the outcome. A code is provided to build and understand the effect of manipulating the leaf scores and the patient number “falling” into each path.

**Results**

**Patient characteristics**

Baseline characteristics of the APACE and TRAPID-AMI per sex are shown in the **Supplemental Table 11**.

**Derivation and Internal validation**

**Triage thresholds**

A FAST-NSTEMI score of <1 for the single hs-cTnT model and of <3 for the serial hs-cTnT model met the prespecified diagnostic performance criteria in the derivation cohort (**Supplemental Table 4**) and triaged 44.9 and 63.8% of patients towards rule-out of NSTEMI. Comparable performance was found in the internal validation cohort, with a sensitivity of 99.7 and 99.6% and an NPV of 99.9 and 99.9%, respectively (**Table 2, Supplemental Figure 7A and C**), and triaged 38.9 and 63.9% of patients towards rule-out of NSTEMI. Slightly higher thresholds triaged an even higher proportion of patients towards rule-out of NSTEMI, albeit at slightly lower sensitivity and NPV.

A FAST-NSTEMI score of 60 or more for the single and serial hs-cTnT models met the prespecified diagnostic performance criteria in the derivation cohort (**Supplemental Table 4**), with a specificity of 97.1 and 98.3%, and triaged 14.7 and 15.9% of patients towards rule-in of NSTEMI. Comparable performance was found in the internal validation cohort with a specificity of 96.1 and 96.4%, and a PPV of 75.6 and 78.7%, respectively (**Table 2, Supplemental Figure 7B and D**). Slightly lower thresholds triaged an even higher proportion of patients towards rule-in of NSTEMI, albeit at slightly lower specificity and PPV.

Direct comparison with the ESC 0/1h-algorithm

Among 1,413 patients eligible for the direct comparison with the ESC 0/1h-algorithm, 231 (16.3%) had NSTEMI (Figure 3 Main Manuscript). FAST-NSTEMI classified more patients than the ESC 0/1h-algorithm to rule out (905 [64.1%] versus 874 [61.8%], respectively) and rule-in (237 [16.8%] versus 213 [15.1%], respectively), leaving fewer patients in the observe zone with FAST-NSTEMI (271 [19.2%] vs 326 [23.1%] p<0.01). FAST-NSTEMI had slightly higher sensitivity, NPV and specificity than the ESC 0/1h-algorithm (sensitivity 99.7% [95% CI, 99.4-100] vs 99.1% [95% CI, 98.6-99.6]; NPV 99.9% [95%CI, 99.7-100] vs 99.8% [95%CI, 99.6%-100%]; Specificity 96.9 % [95% CI, 96.0-97.8] versus 96.2% [95% CI, 95.2-97.2]). In contrast, PPV (74.2% [95% CI, 71.9-76.5]) was lower versus the ESC 0/1h-algorithm (78.9% [95% CI, 76.8 – 81.0]).

**Sensitivity analysis 1: model without time since CPO**

Omission of time since CPO in FAST-NSTEMI still resulted in very high discrimination in both the single and serial hs-cTnT models (0.96 [95% CI: 0.96-0.97] and 0.98 [95% CI: 0.98-0.98], respectively; **Supplemental Figure 10B and E**). Calibration was also comparable for both models (**Supplemental Figure 10 C and F**). A FAST-NSTEMI score of <0.9 for the single hs-cTnT model and of <2 for the serial hs-cTnT model for rule-out (**Supplemental Table 5**), and a FAST-NSTEMI score of more than 60 for both models for rule-in, met our prespecified diagnostic performance criteria in the derivation cohort, resulting in comparable performance in the internal validation cohort (**Supplemental Table 6**). The triage pathway is displayed in the supplemental material **(Supplemental Figure 11).**

**External validation**

**Triage thresholds**

A FAST-NSTEMI score <1 for the single hs-cTnT model and <3 for the serial hs-cTnT model met the prespecified diagnostic performance criteria for the rule-out of NSTEMI in the external cohort, with an NPV of 99.6 and 99.8% and a sensitivity of 99.2 and 99.1%, respectively (**Supplemental Table 8, Supplemental Figure 14 A and C**). A FAST-NSTEMI score of 60 or more for the single and serial hs-cTnT models achieved a specificity of 94.1 and 95.2% and a PPV of 61.9 and 68.3% for the rule-in of NSTEMI, respectively (**Supplemental Table 8, Supplemental Figure 14 B and D**). Thereby, the predefined diagnostic performance criteria for the rule-out of NSTEMI were met only for specificity for the serial hs-cTnT model.

**Sensitivity analysis: Model without CPO**

Discrimination performance of FAST-NSTEMI without time since CPO in external validation was very good, with an AUC for detecting NSTEMI using the single hs-cTnT and serial hs-cTnT model of 0.91 [95% CI: 0.90-0.92] and 0.96 [95% CI: 0.95-0.97] (**Supplemental Figure 15 A and D**), respectively. Calibration curves were similar to the model with CPO (**Supplemental Figure 15 B and E**). Using the derived thresholds, the safety and efficacy for ruling out patients using the single hs-cTnT model decreased compared to the model with CPO, however, precision for ruling in patients increased (**Supplemental Table 10, Supplemental Figure 16**).

**SHAP**

The variables with the strongest impact on the single hs-cTnT model and the serial hs-cTnT model output magnitudes were the 0h hs-cTnT (2.53) and the first serial hs-cTnT (1.7). Time since CPO had a higher impact on the single hs-cTnT model than on the serial hs-cTnT model (0.28 and 2nd highest impact versus 0.12 and 5th highest impact) (**Figure 1**). Sex had no relevant impact in both models. SHAP values can be computed using individual predictions from both models, as shown in (**Supplemental Figure 18A and B**).

1 Koechlin L, Boeddinghaus J, Nestelberger T, *et al.* Performance of the ESC 0/2h-algorithm using high-sensitivity cardiac troponin I in the early diagnosis of myocardial infarction. *Am Heart J* 2021; **242**: 132–7.

2 Thygesen K, Alpert JS, Jaffe AS, *et al.* Fourth Universal Definition of Myocardial Infarction (2018). *Circulation* 2018; **138**. DOI:10.1161/CIR.0000000000000617.

3 Twerenbold R, Costabel JP, Nestelberger T, *et al.* Outcome of Applying the ESC 0/1-hour Algorithm in Patients With Suspected Myocardial Infarction. *J Am Coll Cardiol* 2019; **74**: 483–94.

4 Collins GS, Reitsma JB, Altman DG, Moons KGM. Transparent Reporting of a multivariable prediction model for Individual Prognosis Or Diagnosis (TRIPOD): The TRIPOD Statement. *Ann Intern Med* 2015; **162**: 55–63.

5 Harrell , FE. Regression Modeling Strategies. 2015. DOI:10.1007/978-3-319-19425-7.

6 Lopez-Ayala P, Riley RD, Collins GS, Zimmermann T. Dealing with continuous variables and modelling non-linear associations in healthcare data: practical guide. *BMJ* 2025; **390**. DOI:10.1136/BMJ-2024-082440.

7 Hollmann N, Müller S, Eggensperger K, Hutter F. TabPFN: A Transformer That Solves Small Tabular Classification Problems in a Second. 2022; published online July 5.

8 Guido Van Rossum FLD. Python 3 Reference Manual. Scotts Valley, CA: CreateSpace, 2009.

9 Carpenter J, Bithell J. Bootstrap confidence intervals: when, which, what? A practical guide for medical statisticians. *Stat Med* 2000; **19**: 1141–64.

10 Steyerberg EW, Vickers AJ, Cook NR, *et al.* Assessing the performance of prediction models: a framework for traditional and novel measures. *Epidemiology* 2010; **21**: 128–38.

11 DeLong ER, DeLong DM, Clarke-Pearson DL. Comparing the areas under two or more correlated receiver operating characteristic curves: a nonparametric approach. *Biometrics* 1988; **44**: 837–45.

12 Van Calster B, McLernon DJ, van Smeden M, Wynants L, Steyerberg EW. Calibration: the Achilles heel of predictive analytics. *BMC Med* 2019; **17**: 230.

13 Austin PC, Steyerberg EW. Graphical assessment of internal and external calibration of logistic regression models by using loess smoothers. *Stat Med* 2014; **33**: 517–35.

14 McNemar Q. Note on the Sampling Error of the Difference Between Correlated Proportions or Percentages. *Psychometrika* 1947; **12**: 153–7.

15 R Core Team. R: A Language and Environment for Statistical Computing. 2021.

**Additional ^##^ and contributors to this manuscript to be listed in PUBMED include:**

Carlos Spagnuolo, MD^a^; Jonas Glaeser, MD^a^; Thomas Nestelberger, MD^a,^; Desiree Wussler, MD^a,^; Maria Rubini Gimenez, MD^a,^; Danielle M. Gualandro, MDPhD^a,^; Christian Puelacher, MDPhD^a,^; Jeanne du Fay de Lavallaz, MDPhD^a,^; Julia Reinhardt, PhD^a,^; Kathrin Meissner, SN^a,^; Katharina Rentsch, PhD^a,^; Ksenia Slankamenac, MDPhD Prof.^b^; Beatriz López, MD^c,d^; Gemma Martinez-Nadal^c,d^; Esther Rodriguez Adrada, MD^c,e^; Arnold von Eckardstein, MD^f^; Damian Kawecki, MD^c,g^; Piotr Muzyk, MD^c,g^; Nicolas Geigy, MD^h^; Eliška Potluková, MDPhD^i^; Stephan Steuer, MD^j^. Angelika Hammerer-Lercher, MD^k^; Andreas Buser, MD^l^.

^a^Cardiovascular Research Institute Basel (CRIB) and Department of Cardiology, University Hospital Basel, University of Basel;

^b^ Emergency Department, University Hospital Zürich, University of Zürich; Switzerland;

^c^GREAT network; Rome, Italy;

^d^ Emergency Department, Hospital Clinic, Barcelona, Catalonia, Spain;

^e^ Servicio de Urgencias, Hospital Clínico San Carlos, Madrid, Spain;

^f^Department of Laboratory Medicine, University Hospital Zurich, Switzerland;

^g^2nd Department of Cardiology, School of Medicine with the Division of Dentistry in Zabrze, Medical University of Katowice, Poland.

^h^ Emergency Department, Kantonsspital Basel-Land, Switzerland.

^i^ Department of Internal Medicine, Kantonsspital Basel-Land, Switzerland.

^j^ Emergency Department, Claraspital Basel, Switzerland.

^k^Department of Laboratory Medicine, Hospital Feldkirch, Austria;

^l^Department of Hematology and Blood Bank, University Hospital Basel, Switzerland.

**Additional TRAPID-AMI-Investigators^##^ and contributors to this manuscript to be listed in PUBMED include:**

James McCord, MD^n^; Richard Nowak, MD^o^; Richard Body, MB ChB PhD^p^; Christopher deFilippi, MD^q^; Robert Christenson, MD^r^; Mauro Panteghini, MD^s^; Mario Plebani, MD^t^; Franck Verschuren, PhD^u^; John French, PhD^v^; Silvia Weiser, PhD^w^; Tomas Jernberg, MD^x^; Aitor Alquézar-Arbé, MD, PhD^y^; Jordi Ordonez-Llanos, MD, PhD^z^;

^n^Heart and Vascular Institute, Henry Ford Health System, Detroit, Michigan, United States; ^o^Department of Emergency Medicine, Henry Ford Health System, Detroit, Michigan, United States;

^p^Central Manchester University Hospitals NHS Foundation Trust, Manchester, UK;

^q^Inova Heart and Vascular Institute, Falls Church, Virginia, United States;

^r^University of Maryland School of Medicine, Baltimore, Maryland, United States; Department of Biomedical and Clinical Sciences “Luigi Sacco”,

^s^University of Milan Medical School, Milan, Italy;

^t^Department of Laboratory Medicine, University Hospital of Padova, Padua, Italy;

^u^Department of Acute Medicine, Cliniques Universitaires St-Luc and Universite Catholique de Louvain, Brussels, Belgium;

^v^Liverpool Hospital and University of New South Wales, Liverpool, NSW, Australia;

^w^Roche Diagnostics Germany, Penzberg, Germany;

^x^Department of Medicine, Karolinksa Institutet, Huddinge, Sweden;

^y^Emergency Department, Hospital de la Santa Creu i Sant Pau, Barcelona, Spain;

^z^Department of clinical biochemistry, Universitat Autonoma, Barcelona, Spain.

**Supplemental Tables**

**Supplemental Table 1. Baseline Characteristics of the Upsampled Cohorts for Derivation and Internal Validation of FAST-NSTEMI with Single and Serial Hs-cTnT Measurements**

|  | **Single hs-cTnT model** | | **Serial hs-cTnT model** | |
| --- | --- | --- | --- | --- |
| **Oversampled datasets** | **Training**, N = 19,412*^1^* | **Validation**, N = 4,849*^1^* | **training**, N = 20,442*^1^* | **validation**, N = 5,088*^1^* |
| **Demographics** |  |  |  |  |
| Age | 61 [49, 73] | 62 [49, 74] | 61 [50, 74] | 62 [50, 74] |
| Female | 6,389 (33%) | 1,757 (36%) | 6,549 (32%) | 1,817 (36%) |
| Time since CPO | 5 [2, 15] | 5 [2, 15] | 5 [2, 14] | 5 [2, 14] |
| **Cardiovascular risk factors, n (%)** |  |  |  |  |
| Hypertension | 11,681 (60%) | 2,917 (60%) | 12,568 (62%) | 3,157 (62%) |
| Hypercholesterolemia | 8,135 (49%) | 1,993 (49%) | 8,971 (51%) | 2,183 (50%) |
| Diabetes mellitus | 3,498 (18%) | 926 (19%) | 3,782 (19%) | 1,023 (20%) |
| Current smoker | 5,734 (30%) | 1,461 (30%) | 5,904 (29%) | 1,497 (30%) |
| **History (n%)** |  |  |  |  |
| Previous CAD | 6,566 (34%) | 1,644 (34%) | 7,108 (35%) | 1,764 (35%) |
| Previous MI | 4,567 (24%) | 1,133 (23%) | 4,963 (24%) | 1,200 (24%) |
| Previous Revascularisation | 4,802 (25%) | 1,225 (25%) | 5,288 (26%) | 1,321 (26%) |
| Previous stroke | 861 (5%) | 212 (5%) | 937 (5%) | 225 (5%) |
| **Medications at presentation, n (%)** |  |  |  |  |
| Antiplatelets therapy | 6,753 (35%) | 1,737 (36%) | 7,457 (37%) | 1,898 (37%) |
| Anticoagulants | 2,116 (11%) | 483 (10%) | 2,254 (11%) | 529 (10%) |
| Beta-blockers | 6,658 (34%) | 1,648 (34%) | 7,212 (35%) | 1,788 (35%) |
| Statins | 5,843 (35%) | 1,486 (36%) | 6,473 (37%) | 1,660 (38%) |
| ACEIs/ARBs | 7,528 (39%) | 1,856 (38%) | 8,251 (40%) | 2,059 (41%) |
| **ECG findings, n (%)** |  |  |  |  |
| T-Wave inversion | 1,708 (9%) | 443 (9%) | 1,777 (89%) | 472 (9%) |
| ST depression | 1,492 (8 %) | 358 (7%) | 1,566 (8%) | 373 (7%) |
| Aberration (BBB, pacemaker) | 1,183 (6%) | 308 (6%) | 1,316 (6%) | 342 (7%) |
| None | 15,029 (77%) | 3,740 (77%) | 15,783 (77%) | 3,901 (77%) |
| **Laboratory findings, median (IQR)** |  |  |  |  |
| eGFR | 87 [70, 100] | 87 [70, 102] | 86 [69, 100] | 86 [70, 101] |
| **Troponin, median (IQR)** |  |  |  |  |
| Hs-cTnT 0h | 8 [4, 19] | 8 [4, 19] | 8 [4, 19] | 9 [4, 19] |
| Hs-cTnT 1h | 8 [4, 20] | 8 [4, 20] | 8 [4, 21] | 9 [4, 21] |
| Hs-cTnT 2h | 8 [4, 21] | 8 [4, 21] | 8 [4, 22] | 9 [4, 22] |
| Hs-cTnT 3h | 9 [5, 25] | 10 [5, 26] | 9 [5, 24] | 10 [5, 26] |
| First serial Hs-cTnT | 8 [4, 20] | 8 [4, 20] | 8 [4, 21] | 9 [4, 21] |

*^1^*Median (Q1, Q3); n (%)CAD = coronary artery disease; MI = myocardial infarction; ECG = Electrocardiogram; BBB=Bundle branch block; eGFR = estimated glomerular filtration rate; ACEIs/ARBs = Angiotensin-converting enzyme inhibitors or angiotensin receptor blocker; hs-cTnT = high-sensitivity cardiac troponin T

| **Supplemental Table 2. Baseline Characteristics of the APACE cohort per center** | | | | | | | | | | | | | |
| --- | --- | --- | --- | --- | --- | --- | --- | --- | --- | --- | --- | --- | --- |
| **APACE** | **Basel** N = 5,482*^1^* | **Olten** N = 387*^1^* | **Limmattal** N = 131*^1^* | **Luzern** N = 10*^1^* | **Bruderholz** N = 29*^1^* | **Barcelona_del_Mar** N = 588*^1^* | **Barcelona_Clinic** N = 899*^1^* | **Brno** N = 95*^1^* | **Zabre** N = 262*^1^* | **Zuerich** N = 278*^1^* | **Liestal** N = 30*^1^* | **Madrid** N = 49*^1^* | **LUKS/Luzern** N = 27*^1^* |
| **Demographics** | | | | | | | | | | | | | |
| Age | 60 (47, 73) | 61 (50, 74) | 58 (50, 71) | 58 (53, 65) | 64 (46, 74) | 67 (54, 78) | 65 (52, 76) | 63 (51, 71) | 64 (57, 71) | 56 (43, 69) | 59 (46, 69) | 64 (54, 75) | 68 (57, 77) |
| female | 1,781 (32%) | 133 (34%) | 44 (34%) | 3 (30%) | 12 (41%) | 213 (36%) | 354 (39%) | 30 (32%) | 93 (35%) | 81 (29%) | 13 (43%) | 23 (47%) | 8 (30%) |
| Time since CPO | 6 (3, 23) | 4 (2, 12) | 4 (2, 8) | 5 (2, 36) | 4 (2, 8) | 4 (2, 7) | 4 (2, 11) | 4 (2, 7) | 6 (4, 8) | 15 (4, 72) | 17 (5, 62) | 4 (2, 8) | 67 (9, 72) |
| **Cardiovascular risk factors, n (%)** | | | | | | | | | | | | | |
| Previous CAD | 1,703 (31%) | 122 (32%) | 26 (20%) | 3 (30%) | 5 (17%) | 215 (37%) | 300 (33%) | 27 (28%) | 91 (35%) | 74 (27%) | 6 (20%) | 17 (35%) | 10 (37%) |
| Previous MI | 1,208 (22%) | 79 (20%) | 21 (16%) | 3 (30%) | 3 (10%) | 150 (26%) | 235 (26%) | 21 (22%) | 61 (23%) | 51 (18%) | 3 (10%) | 16 (33%) | 9 (33%) |
| Previous Revascularisation | 1,458 (27%) | 100 (26%) | 19 (15%) | 2 (20%) | 4 (14%) | 149 (25%) | 241 (27%) | 23 (24%) | 85 (32%) | 66 (24%) | 5 (17%) | 15 (31%) | 9 (33%) |
| Previous stroke | 284 (5.2%) | 23 (5.9%) | 3 (2.3%) | 0 (0%) | 2 (6.9%) | 40 (6.8%) | 40 (4.4%) | 4 (4.2%) | 17 (6.5%) | 7 (2.5%) | 0 (0%) | 5 (10%) | 4 (15%) |
| **History, n (%)** | | | | | | | | | | | | | |
| Hypertension | 3,132 (57%) | 235 (61%) | 76 (58%) | 7 (70%) | 16 (55%) | 421 (72%) | 564 (63%) | 61 (64%) | 201 (77%) | 136 (49%) | 14 (47%) | 33 (67%) | 13 (48%) |
| Dyslipidemia | 2,404 (44%) | 215 (56%) | 65 (50%) | 6 (60%) | 12 (41%) | 372 (63%) | 515 (57%) | 44 (46%) | 178 (68%) | 129 (46%) | 9 (30%) | 36 (73%) | 15 (56%) |
| Diabetes | 840 (15%) | 64 (17%) | 30 (23%) | 1 (10%) | 2 (6.9%) | 171 (29%) | 215 (24%) | 20 (21%) | 66 (25%) | 31 (11%) | 5 (17%) | 14 (29%) | 8 (30%) |
| Active smoker | 1,415 (26%) | 113 (29%) | 44 (34%) | 3 (30%) | 12 (41%) | 118 (20%) | 159 (18%) | 35 (37%) | 56 (22%) | 82 (30%) | 7 (23%) | 8 (16%) | 6 (22%) |
| **ECG findings, n (%)** | | | | | | | | | | | | | |
| T wave inversion | 460 (8.5%) | 38 (9.9%) | 7 (5.5%) | 4 (40%) | 0 (0%) | 57 (9.8%) | 68 (7.6%) | 15 (16%) | 36 (14%) | 24 (8.7%) | 3 (10%) | 4 (9.1%) | 4 (15%) |
| ST depression | 421 (7.8%) | 50 (13%) | 17 (13%) | 3 (30%) | 6 (21%) | 81 (14%) | 69 (7.7%) | 24 (25%) | 73 (29%) | 20 (7.2%) | 2 (6.7%) | 4 (9.1%) | 3 (11%) |
| Aberration (BBB, pacemaker) | 282 (5.2%) | 25 (6.5%) | 2 (1.6%) | 0 (0%) | 1 (3.6%) | 58 (10.0%) | 57 (6.4%) | 6 (6.3%) | 14 (5.6%) | 19 (6.9%) | 0 (0%) | 3 (6.8%) | 1 (3.7%) |
| None | 4,252 (79%) | 269 (70%) | 102 (80%) | 3 (30%) | 21 (75%) | 386 (66%) | 700 (78%) | 50 (53%) | 129 (51%) | 213 (77%) | 25 (83%) | 33 (75%) | 19 (70%) |
| **Medications at presentation, n (%)** | | | | | | | | | | | | | |
| Anticoagulants | 538 (9.8%) | 37 (9.6%) | 6 (4.6%) | 1 (10%) | 0 (0%) | 75 (13%) | 102 (11%) | 5 (5.3%) | 18 (6.9%) | 15 (5.4%) | 1 (3.3%) | 6 (12%) | 1 (3.7%) |
| Beta-blockers | 1,784 (33%) | 120 (31%) | 36 (27%) | 6 (60%) | 6 (21%) | 210 (36%) | 303 (34%) | 28 (29%) | 114 (44%) | 70 (25%) | 10 (33%) | 18 (37%) | 10 (37%) |
| Statins | 1,740 (32%) | 139 (36%) | 29 (22%) | 4 (40%) | 3 (10%) | 292 (50%) | 341 (38%) | 27 (28%) | 126 (48%) | 90 (32%) | 8 (27%) | 30 (61%) | 14 (52%) |
| **Laboratory findings, median (IQR)** | | | | | | | | | | | | | |
| eGFR | 88 (71, 102) | 85 (65, 100) | 90 (76, 102) | 84 (79, 101) | 96 (76, 111) | 84 (65, 98) | 85 (67, 100) | 81 (66, 94) | 83 (69, 96) | 88 (72, 102) | 88 (68, 97) | 88 (76, 99) | 83 (55, 95) |
| **Troponin, median (IQR)** | | | | | | | | | | | | | |
| Hs-cTnT 0h | 8 (4, 21) | 9 (5, 26) | 8 (5, 17) | 9 (5, 223) | 7 (4, 15) | 10 (5, 23) | 8 (4, 20) | 41 (13, 178) | 52 (16, 240) | 5 (3, 15) | 5 (3, 15) | 7 (4, 12) | 28 (6, 85) |
| Hs-cTnT 1h | 8 (4, 20) | 9 (5, 25) | 8 (5, 14) | 8 (8, 8) | 6 (3, 19) | 11 (5, 24) | 8 (4, 19) | 79 (15, 499) | 84 (18, 405) | 5 (3, 16) | 5 (3, 18) | 8 (5, 15) | 27 (6, 86) |
| Hs-cTnT 2h | 8 (5, 21) | 9 (5, 24) | 8 (5, 14) | 3 (3, 3) | 9 (4, 24) | 11 (6, 24) | 9 (4, 21) | 109 (13, 566) | 113 (21, 473) | 6 (3, 16) | 4 (3, 21) | 8 (5, 15) | 24 (10, 191) |
| Hs-cTnT 3h | 10 (5, 26) | 10 (6, 50) | 7 (5, 11) | 8 (8, 8) | 25 (10, 37) | 9 (4, 24) | 9 (4, 22) | 115 (13, 615) | 119 (19, 610) | 10 (4, 25) | 6 (3, 27) | 8 (4, 13) | 24 (9, 209) |
| First consecutive Hs-cTnT | 8 (4, 21) | 9 (5, 26) | 8 (5, 14) | 8 (8, 8) | 6 (3, 19) | 11 (5, 24) | 8 (4, 20) | 79 (15, 499) | 85 (17, 410) | 6 (3, 16) | 4 (3, 15) | 8 (5, 15) | 27 (9, 146) |
| *^1^*Median (Q1, Q3); n (%)CAD = coronary artery disease; MI = myocardial infarction; ECG = Electrocardiogram; BBB=Bundle branch block; eGFR = estimated glomerular filtration rate; ACEIs/ARBs = Angiotensin-converting enzyme inhibitors or angiotensin receptor blocker; hs-cTnT = high-sensitivity cardiac troponin T | | | | | | | | | | | | | |
|  | | | | | | | | | | | | | |

| **Supplemental Table 3. Baseline Characteristics of the TRAPID-AMI cohort per center** | | | | | | | | | | | | |
| --- | --- | --- | --- | --- | --- | --- | --- | --- | --- | --- | --- | --- |
| **TRAPID-AMI** | **Louvain (Belgium)** N = 80*^1^* | **Brussels (Belgium)** N = 73*^1^* | **Basel (Switzerland)** N = 107*^1^* | **Heidelberg (Germany)** N = 127*^1^* | **Nuremberg (Germany)** N = 134*^1^* | **Barcelona (Spain)** N = 113*^1^* | **Manchester (UK)** N = 110*^1^* | **Milan (Italy)** N = 114*^1^* | **Padua (Italy)** N = 84*^1^* | **Sesto San Giovanni (Italy)** N = 98*^1^* | **Baltimore (USA)** N = 112*^1^* | **Detroit (USA)** N = 130*^1^* |
| **Demographics** | | | | | | | | | | | | |
| Age | 57 (47, 67) | 55 (48, 72) | 58 (43, 70) | 65 (56, 75) | 64 (53, 76) | 71 (57, 79) | 59 (51, 71) | 69 (55, 77) | 66 (53, 74) | 69 (57, 77) | 56 (48, 66) | 54 (47, 62) |
| female | 22 (28%) | 30 (41%) | 28 (26%) | 38 (30%) | 52 (39%) | 42 (37%) | 39 (35%) | 42 (37%) | 28 (33%) | 28 (29%) | 63 (56%) | 65 (50%) |
| Time since CPO | 3 (1, 4) | 3 (1, 5) | 3 (2, 6) | 5 (2, 10) | 3 (2, 5) | 2 (1, 4) | 2 (1, 3) | 3 (1, 14) | 2 (1, 4) | 3 (2, 5) | 4 (1, 8) | 3 (2, 8) |
| **Cardiovascular risk factors, n (%)** | | | | | | | | | | | | |
| Previous CAD | 35 (44%) | 21 (29%) | 30 (28%) | 64 (50%) | 89 (67%) | 53 (47%) | 53 (51%) | 44 (39%) | 23 (28%) | 45 (46%) | 36 (32%) | 37 (28%) |
| Previous MI | 25 (31%) | 17 (23%) | 15 (14%) | 28 (22%) | 33 (25%) | 30 (27%) | 38 (36%) | 37 (33%) | 17 (21%) | 34 (35%) | 14 (13%) | 31 (24%) |
| Previous Revascularisation | 6 (7.5%) | 6 (8.2%) | 11 (10%) | 34 (27%) | 22 (16%) | 18 (16%) | 11 (10%) | 19 (17%) | 12 (14%) | 16 (16%) | 4 (3.6%) | 2 (1.5%) |
| Previous stroke | 0 (NA%) | 0 (NA%) | 0 (NA%) | 0 (NA%) | 0 (NA%) | 0 (NA%) | 0 (NA%) | 0 (NA%) | 0 (NA%) | 0 (NA%) | 0 (NA%) | 0 (NA%) |
| **History, n (%)** | | | | | | | | | | | | |
| Hypertension | 50 (63%) | 36 (49%) | 58 (55%) | 98 (77%) | 102 (77%) | 78 (69%) | 53 (52%) | 64 (56%) | 52 (62%) | 51 (52%) | 61 (54%) | 102 (78%) |
| Dyslipidemia | 0 (NA%) | 0 (NA%) | 0 (NA%) | 0 (NA%) | 0 (NA%) | 0 (NA%) | 0 (NA%) | 0 (NA%) | 0 (NA%) | 0 (NA%) | 0 (NA%) | 0 (NA%) |
| Diabetes | 15 (19%) | 16 (22%) | 17 (16%) | 26 (20%) | 36 (27%) | 28 (25%) | 27 (28%) | 23 (20%) | 14 (17%) | 20 (20%) | 21 (19%) | 27 (21%) |
| Active smoker | 50 (63%) | 40 (56%) | 71 (66%) | 83 (65%) | 81 (60%) | 73 (65%) | 54 (56%) | 59 (52%) | 50 (60%) | 51 (55%) | 62 (55%) | 82 (63%) |
| **ECG findings, n (%)** | | | | | | | | | | | | |
| None | 67 (84%) | 62 (85%) | 83 (78%) | 90 (71%) | 89 (66%) | 77 (68%) | 78 (71%) | 76 (67%) | 57 (68%) | 67 (68%) | 92 (82%) | 98 (75%) |
| T wave inversion | 5 (6.3%) | 4 (5.5%) | 13 (12%) | 13 (10%) | 29 (22%) | 11 (9.7%) | 18 (16%) | 25 (22%) | 10 (12%) | 16 (16%) | 3 (2.7%) | 23 (18%) |
| ST depression | 4 (5.0%) | 4 (5.5%) | 6 (5.6%) | 9 (7.1%) | 10 (7.5%) | 12 (11%) | 6 (5.5%) | 4 (3.5%) | 12 (14%) | 5 (5.1%) | 10 (8.9%) | 3 (2.3%) |
| Aberration (BBB, pacemaker) | 4 (5.0%) | 3 (4.1%) | 5 (4.7%) | 15 (12%) | 6 (4.5%) | 13 (12%) | 8 (7.3%) | 9 (7.9%) | 5 (6.0%) | 10 (10%) | 7 (6.3%) | 6 (4.6%) |
| **Medications at presentation, n (%)** | | | | | | | | | | | | |
| Anticoagulants | 4 (5.0%) | 7 (9.6%) | 7 (6.5%) | 56 (44%) | 44 (33%) | 17 (15%) | 6 (6.6%) | 14 (12%) | 8 (9.6%) | 13 (13%) | 11 (9.8%) | 6 (4.6%) |
| Beta-blockers | 26 (33%) | 21 (29%) | 32 (30%) | 73 (57%) | 71 (53%) | 35 (31%) | 38 (40%) | 39 (35%) | 16 (20%) | 54 (55%) | 36 (32%) | 47 (36%) |
| Statins | 0 (NA%) | 0 (NA%) | 0 (NA%) | 0 (NA%) | 0 (NA%) | 0 (NA%) | 0 (NA%) | 0 (NA%) | 0 (NA%) | 0 (NA%) | 0 (NA%) | 0 (NA%) |
| **Laboratory findings, median (IQR)** | | | | | | | | | | | | |
| eGFR | 81 (68, 100) | 84 (72, 97) | 86 (69, 100) | 90 (71, 106) | 77 (61, 94) | 81 (64, 95) | 77 (63, 91) | 86 (70, 102) | 67 (58, 81) | 79 (65, 94) | 72 (61, 87) | 87 (71, 104) |
| **Troponin, median (IQR)** | | | | | | | | | | | | |
| Hs-cTnT 0h | 4 (3, 9) | 4 (3, 14) | 4 (3, 17) | 10 (3, 25) | 9 (3, 27) | 11 (3, 23) | 5 (3, 13) | 8 (3, 19) | 6 (3, 14) | 10 (5, 27) | 3 (3, 10) | 5 (3, 11) |
| Hs-cTnT 1h | 4 (3, 9) | 4 (3, 12) | 4 (3, 16) | 10 (3, 26) | 9 (3, 28) | 11 (4, 21) | 4 (3, 13) | 8 (3, 19) | 6 (3, 14) | 11 (5, 24) | 4 (3, 10) | 5 (3, 12) |
| Hs-cTnT 2h | 4 (3, 10) | 4 (3, 16) | 4 (3, 13) | 9 (3, 27) | 9 (4, 32) | 10 (4, 23) | 5 (3, 11) | 7 (3, 20) | 8 (4, 18) | 11 (5, 26) | 3 (3, 11) | 5 (3, 11) |
| Hs-cTnT 3h | 0 (NA%) | 0 (NA%) | 0 (NA%) | 0 (NA%) | 0 (NA%) | 0 (NA%) | 0 (NA%) | 0 (NA%) | 0 (NA%) | 0 (NA%) | 0 (NA%) | 0 (NA%) |
| First consecutive Hs-cTnT | 4 (3, 9) | 4 (3, 12) | 4 (3, 16) | 10 (3, 26) | 9 (3, 28) | 11 (4, 21) | 4 (3, 13) | 8 (3, 19) | 6 (3, 14) | 11 (5, 24) | 4 (3, 10) | 5 (3, 12) |
| *^1^*Median (Q1, Q3); n (%)CAD = coronary artery disease; MI = myocardial infarction; ECG = Electrocardiogram; BBB=Bundle branch block; eGFR = estimated glomerular filtration rate; ACEIs/ARBs = Angiotensin-converting enzyme inhibitors or angiotensin receptor blocker; hs-cTnT = high-sensitivity cardiac troponin T | | | | | | | | | | | | |

**Supplemental Table 4. Diagnostic thresholds of FAST-NSTEMI in the derivation cohorts**

|  | **Ruling-in thresholds and metrics** | | | | | |
| --- | --- | --- | --- | --- | --- | --- |
|  | **Single hs-cTnT model** | | | **Serial hs-cTnT model** | | |
| **Threshold (%)** | **Specificity** | **PPV** | **Proportion RI (%)*^1^*** | **Specificity** | **PPV** | **Proportion RI (%)*^1^*** |
| 50 | 0.9579 | 0.7987 | 17.15 | 0.9764 | 0.8898 | 17.44 |
| 55 | 0.9653 | 0.8211 | 15.90 | 0.9801 | 0.9022 | 16.61 |
| 60 | 0.9710 | 0.8389 | 14.74 | 0.9829 | 0.9121 | 15.85 |
| 65 | 0.9762 | 0.8570 | 13.62 | 0.9857 | 0.9226 | 15.05 |
| 70 | 0.9812 | 0.8769 | 12.51 | 0.9892 | 0.9375 | 14.10 |
| 75 | 0.9852 | 0.8932 | 11.38 | 0.9921 | 0.9508 | 13.13 |
| 80 | 0.9901 | 0.9166 | 9.70 | 0.9940 | 0.9591 | 11.97 |

|  | **Ruling-out thresholds and metrics** | | | | | |
| --- | --- | --- | --- | --- | --- | --- |
|  | **Single hs-cTnT model** | | | **Serial hs-cTnT model** | | |
| **Threshold (%)** | **Sensitivity** | **NPV** | **Proportion RO (%)*^1^*** | **Sensitivity** | **NPV** | **Proportion RO (%)*^1^*** |
| 1.0 | 0.9949 | 0.9979 | 44.87 | 0.9942 | 0.9981 | 55.85 |
| 1.5 | 0.9926 | 0.9973 | 50.37 | 0.9934 | 0.9979 | 59.04 |
| 2.0 | 0.9877 | 0.9959 | 54.23 | 0.9931 | 0.9979 | 60.91 |
| 2.5 | 0.9851 | 0.9953 | 56.82 | 0.9918 | 0.9976 | 62.54 |
| 3.0 | 0.9840 | 0.9951 | 58.82 | 0.9912 | 0.9975 | 63.80 |
| 3.5 | 0.9814 | 0.9944 | 60.23 | 0.9907 | 0.9974 | 64.96 |
| 4.0 | 0.9803 | 0.9942 | 61.13 | 0.9899 | 0.9972 | 65.85 |

**Supplemental Table 5. Diagnostic thresholds of FAST-NSTEMI without time since onset of chest pain in the derivation cohorts**

|  | **Ruling-in thresholds and metrics** | | | | | |
| --- | --- | --- | --- | --- | --- | --- |
|  | **Single hs-cTnT model** | | | **Serial hs-cTnT model** | | |
| **Threshold (%)** | **Specificity** | **PPV** | **Proportion RI (%)*^1^*** | **Specificity** | **PPV** | **Proportion RI (%)*^1^*** |
| 50 | 0.9587 | 0.7921 | 16.32 | 0.9655 | 0.8418 | 17.85 |
| 55 | 0.9643 | 0.8092 | 15.35 | 0.9699 | 0.8551 | 17.00 |
| 60 | 0.9691 | 0.8211 | 14.20 | 0.9751 | 0.8731 | 16.04 |
| 65 | 0.9757 | 0.8459 | 12.93 | 0.9794 | 0.8887 | 15.16 |
| 70 | 0.9803 | 0.8647 | 11.97 | 0.9831 | 0.9021 | 14.14 |
| 75 | 0.9857 | 0.8875 | 10.45 | 0.9869 | 0.9179 | 13.03 |
| 80 | 0.9890 | 0.8944 | 8.54 | 0.9904 | 0.9325 | 11.67 |

|  | **Ruling-out thresholds and metrics** | | | | | |
| --- | --- | --- | --- | --- | --- | --- |
|  | **Single hs-cTnT model** | | | **Serial hs-cTnT model** | | |
| **Threshold (%)** | **Sensitivity** | **NPV** | **Proportion RO (%)*^1^*** | **Sensitivity** | **NPV** | **Proportion RO (%)*^1^*** |
| 0.85 | 0.9939 | 0.9974 | 42.67 | 0.9936 | 0.9979 | 55.94 |
| 0.90 | 0.9933 | 0.9973 | 44.73 | 0.9936 | 0.9979 | 56.62 |
| 0.95 | 0.9916 | 0.9968 | 46.21 | 0.9936 | 0.9980 | 57.15 |
| 1.00 | 0.9914 | 0.9967 | 47.49 | 0.9936 | 0.9980 | 57.56 |
| 1.50 | 0.9889 | 0.9961 | 50.82 | 0.9920 | 0.9975 | 59.41 |
| 2.00 | 0.9855 | 0.9951 | 52.69 | 0.9917 | 0.9975 | 61.02 |
| 2.50 | 0.9833 | 0.9946 | 55.31 | 0.9906 | 0.9973 | 62.24 |

AUC = area under the curve; NPV = negative predictive value; PPV = positive predictive value; RO = rule-out; RI = rule-in

**Supplemental Table 6. Diagnostic thresholds of FAST-NSTEMI without time since onset of chest pain in the internal validation cohorts**

|  | **Ruling-in thresholds and metrics** | | | | | |
| --- | --- | --- | --- | --- | --- | --- |
|  | **Single hs-cTnT model** | | | **Serial hs-cTnT model** | | |
| **Threshold (%)** | **Specificity** | **PPV** | **Proportion RI (%)*^1^*** | **Specificity** | **PPV** | **Proportion RI (%)*^1^*** |
| 50 | 0.9594 | 0.7937 | 16.16 | 0.9602 | 0.8155 | 17.77 |
| 55 | 0.9615 | 0.7971 | 15.59 | 0.9673 | 0.8386 | 16.66 |
| 60 | 0.9683 | 0.8167 | 14.18 | 0.9745 | 0.8672 | 15.80 |
| 65 | 0.9711 | 0.8235 | 13.45 | 0.9769 | 0.8728 | 14.95 |
| 70 | 0.9773 | 0.8479 | 12.26 | 0.9841 | 0.9052 | 13.84 |
| 75 | 0.9828 | 0.8744 | 11.24 | 0.9865 | 0.9110 | 12.52 |
| 80 | 0.9876 | 0.8889 | 9.15 | 0.9904 | 0.9322 | 11.61 |

|  | **Ruling-out thresholds and metrics** | | | | | |
| --- | --- | --- | --- | --- | --- | --- |
|  | **Single hs-cTnT model** | | | **Serial hs-cTnT model** | | |
| **Threshold (%)** | **Sensitivity** | **NPV** | **Proportion RO (%)*^1^*** | **Sensitivity** | **NPV** | **Proportion RO (%)*^1^*** |
| 0.85 | 0.9905 | 0.9961 | 43.16 | 1.0000 | 1.0000 | 55.74 |
| 0.90 | 0.9905 | 0.9963 | 45.25 | 1.0000 | 1.0000 | 56.66 |
| 0.95 | 0.9905 | 0.9964 | 46.55 | 1.0000 | 1.0000 | 56.92 |
| 1.00 | 0.9874 | 0.9953 | 48.02 | 1.0000 | 1.0000 | 57.51 |
| 1.50 | 0.9874 | 0.9956 | 51.53 | 1.0000 | 1.0000 | 59.54 |
| 2.00 | 0.9811 | 0.9937 | 53.50 | 1.0000 | 1.0000 | 61.38 |
| 2.50 | 0.9811 | 0.9939 | 55.76 | 0.9926 | 0.9979 | 62.75 |

AUC = area under the curve; NPV = negative predictive value; PPV = positive predictive value; RO = rule-out; RI = rule-in

**Supplemental Table 7. Diagnostic thresholds of FAST-NSTEMI using the central adjudication based on sex specific URLs as the reference in the internal validation cohort**

|  | **Ruling-in thresholds and metrics** | | | | | |
| --- | --- | --- | --- | --- | --- | --- |
|  | **Single hs-cTnT model** | | | **Serial hs-cTnT model** | | |
| **Threshold (%)** | **Specificity** | **PPV** | **Proportion RI (%)*^1^*** | **Specificity** | **PPV** | **Proportion RI (%)*^1^*** |
| 50 | 0.9481 | 0.7311 | 15.92 | 0.9544 | 0.7761 | 16.75 |
| 55 | 0.9554 | 0.7534 | 14.92 | 0.9594 | 0.7884 | 15.75 |
| 60 | 0.9610 | 0.7692 | 13.91 | 0.9625 | 0.7967 | 15.17 |
| 65 | 0.9659 | 0.7824 | 12.91 | 0.9665 | 0.8092 | 14.42 |
| 70 | 0.9716 | 0.7953 | 11.44 | 0.9716 | 0.8272 | 13.50 |
| 75 | 0.9765 | 0.8000 | 9.70 | 0.9767 | 0.8425 | 12.17 |
| 80 | 0.9838 | 0.8413 | 8.43 | 0.9797 | 0.8400 | 10.42 |

|  | **Ruling-out thresholds and metrics** | | | | | |
| --- | --- | --- | --- | --- | --- | --- |
|  | **Single hs-cTnT model** | | | **Serial hs-cTnT model** | | |
| **Threshold (%)** | **Sensitivity** | **NPV** | **Proportion RO (%)*^1^*** | **Sensitivity** | **NPV** | **Proportion RO (%)*^1^*** |
| 1.0 | 0.9962 | 0.9983 | 38.80 | 0.9953 | 0.9985 | 55.75 |
| 1.5 | 0.9772 | 0.9915 | 47.29 | 0.9907 | 0.9972 | 59.75 |
| 2.0 | 0.9658 | 0.9887 | 53.38 | 0.9907 | 0.9973 | 61.92 |
| 2.5 | 0.9658 | 0.9893 | 56.05 | 0.9860 | 0.9961 | 63.33 |
| 3.0 | 0.9620 | 0.9885 | 58.06 | 0.9860 | 0.9961 | 64.75 |
| 3.5 | 0.9506 | 0.9854 | 59.53 | 0.9860 | 0.9962 | 65.50 |
| 4.0 | 0.9468 | 0.9846 | 60.74 | 0.9860 | 0.9962 | 66.17 |

AUC = area under the curve; NPV = negative predictive value; PPV = positive predictive value; RO = rule-out; RI = rule-in

**Supplemental Table 8. Diagnostic thresholds of FAST-NSTEMI in the external validation cohorts**

|  | **Ruling-in thresholds and metrics** | | | | | |
| --- | --- | --- | --- | --- | --- | --- |
|  | **Single hs-cTnT model** | | | **Serial hs-cTnT model** | | |
| **Threshold (%)** | **Specificity** | **PPV** | **Proportion RI (%)*^1^*** | **Specificity** | **PPV** | **Proportion RI (%)*^1^*** |
| 50 | 0.9179 | 0.5613 | 16.20 | 0.9372 | 0.6357 | 15.20 |
| 55 | 0.9285 | 0.5857 | 14.93 | 0.9473 | 0.6667 | 13.94 |
| 60 | 0.9406 | 0.6191 | 13.50 | 0.9520 | 0.6836 | 13.37 |
| 65 | 0.9508 | 0.6481 | 12.11 | 0.9597 | 0.7167 | 12.53 |
| 70 | 0.9614 | 0.6877 | 10.69 | 0.9645 | 0.7285 | 11.54 |
| 75 | 0.9671 | 0.7049 | 9.65 | 0.9710 | 0.7394 | 9.82 |
| 80 | 0.9744 | 0.7313 | 8.23 | 0.9793 | 0.7727 | 8.04 |

|  | **Ruling-out thresholds and metrics** | | | | | |
| --- | --- | --- | --- | --- | --- | --- |
|  | **Single hs-cTnT model** | | | **Serial hs-cTnT model** | | |
| **Threshold (%)** | **Sensitivity** | **NPV** | **Proportion RO (%)*^1^*** | **Sensitivity** | **NPV** | **Proportion RO (%)*^1^*** |
| 1.0 | 0.9924 | 0.9963 | 27.94 | 0.9956 | 0.9989 | 47.68 |
| 1.5 | 0.9863 | 0.9946 | 33.84 | 0.9956 | 0.9990 | 53.73 |
| 2.0 | 0.9771 | 0.9929 | 43.08 | 0.9912 | 0.9981 | 56.45 |
| 2.5 | 0.9710 | 0.9921 | 48.98 | 0.9912 | 0.9982 | 58.85 |
| 3.0 | 0.9649 | 0.9910 | 52.25 | 0.9912 | 0.9983 | 61.31 |
| 3.5 | 0.9619 | 0.9905 | 53.69 | 0.9912 | 0.9983 | 62.92 |
| 4.0 | 0.9619 | 0.9906 | 54.69 | 0.9868 | 0.9975 | 63.92 |

**Supplemental Table 9. Diagnostic thresholds of FAST-NSTEMI in the external validation cohorts with liberal imputation of the time since onset of chest pain**

|  | **Ruling-in thresholds and metrics** | | | | | |
| --- | --- | --- | --- | --- | --- | --- |
|  | **Single hs-cTnT model** | | | **Serial hs-cTnT model** | | |
| **Threshold (%)** | **Specificity** | **PPV** | **Proportion RI (%)*^1^*** | **Specificity** | **PPV** | **Proportion RI (%)*^1^*** |
| 50 | 0. 9271 | 0. 5832 | 15.14 | 0.9461 | 0. 6667 | 14.26 |
| 55 | 0. 9368 | 0. 6027 | 13.76 | 0. 9520 | 0. 6848 | 13.42 |
| 60 | 0. 9477 | 0. 6389 | 12.54 | 0. 9562 | 0. 6967 | 12.74 |
| 65 | 0. 9555 | 0. 6631 | 11.43 | 0. 9621 | 0. 7205 | 11.96 |
| 70 | 0. 9640 | 0. 6948 | 10.20 | 0. 9710 | 0. 7538 | 10.39 |
| 75 | 0. 9699 | 0. 7152 | 9.14 | 0. 9751 | 0. 7586 | 9.09 |
| 80 | 0. 9768 | 0. 7315 | 7.48 | 0. 9805 | 0. 7770 | 7.73 |

|  | **Ruling-out thresholds and metrics** | | | | | |
| --- | --- | --- | --- | --- | --- | --- |
|  | **Single hs-cTnT model** | | | **Serial hs-cTnT model** | | |
| **Threshold (%)** | **Sensitivity** | **NPV** | **Proportion RO (%)*^1^*** | **Sensitivity** | **NPV** | **Proportion RO (%)*^1^*** |
| 1.0 | 0. 9848 | 0. 9938 | 32.96 | 0. 9956 | 0. 9989 | 48.56 |
| 1.5 | 0. 9710 | 0. 9907 | 41.83 | 0. 9956 | 0. 9990 | 54.41 |
| 2.0 | 0. 9680 | 0. 9910 | 47.85 | 0. 9912 | 0. 9982 | 57.13 |
| 2.5 | 0. 9649 | 0. 9909 | 51.62 | 0. 9912 | 0. 9982 | 59.58 |
| 3.0 | 0. 9634 | 0. 9909 | 53.79 | 0. 9912 | 0. 9983 | 62.14 |
| 3.5 | 0. 9588 | 0. 9900 | 55.22 | 0. 9912 | 0. 9984 | 63.45 |
| 4.0 | 0. 9573 | 0. 9898 | 56.02 | 0. 9868 | 0. 9976 | 64.28 |

**Supplemental Table 10. Diagnostic thresholds of FAST-NSTEMI without time since the onset of chest pain in the external validation cohorts**

|  | **Ruling-in thresholds and metrics** | | | | | |
| --- | --- | --- | --- | --- | --- | --- |
|  | **Single hs-cTnT model** | | | **Serial hs-cTnT model** | | |
| **Threshold (%)** | **Specificity** | **PPV** | **Proportion RI (%)*^1^*** | **Specificity** | **PPV** | **Proportion RI (%)*^1^*** |
| 50 | 0.9333 | 0.5931 | 14.20 | 0.9396 | 0.6507 | 15.25 |
| 55 | 0.9416 | 0.6147 | 13.13 | 0.9455 | 0.6679 | 14.46 |
| 60 | 0.9510 | 0.6443 | 11.92 | 0.9526 | 0.6958 | 13.73 |
| 65 | 0.9612 | 0.6852 | 10.67 | 0.9585 | 0.7095 | 12.58 |
| 70 | 0.9676 | 0.7110 | 9.71 | 0.9645 | 0.7273 | 11.49 |
| 75 | 0.9726 | 0.7251 | 8.64 | 0.9710 | 0.7538 | 10.39 |
| 80 | 0.9806 | 0.7567 | 6.90 | 0.9757 | 0.7760 | 9.56 |

|  | **Ruling-out thresholds and metrics** | | | | | |
| --- | --- | --- | --- | --- | --- | --- |
|  | **Single hs-cTnT model** | | | **Serial hs-cTnT model** | | |
| **Threshold (%)** | **Sensitivity** | **NPV** | **Proportion RO (%)*^1^*** | **Sensitivity** | **NPV** | **Proportion RO (%)*^1^*** |
| 0.85 | 0.9878 | 0.9954 | 35.25 | 0.9956 | 0.9989 | 49.09 |
| 0.90 | 0.9817 | 0.9937 | 39.16 | 0.9956 | 0.9990 | 50.34 |
| 0.95 | 0.9771 | 0.9926 | 41.60 | 0.9956 | 0.9990 | 51.33 |
| 1.00 | 0.9756 | 0.9924 | 43.24 | 0.9956 | 0.9990 | 51.91 |
| 1.50 | 0.9695 | 0.9915 | 48.18 | 0.9956 | 0.9991 | 55.56 |
| 2.00 | 0.9649 | 0.9905 | 49.73 | 0.9912 | 0.9982 | 58.17 |
| 2.50 | 0.9619 | 0.9902 | 52.31 | 0.9912 | 0.9983 | 60.63 |

| **Supplemental Table 11.** **Baseline characteristics of the APACE and TRAPID-AMI cohort per sex** | | |
| --- | --- | --- |
| **APACE and TRAPID-AMI** | **male** | **female** |
|  | **N = 6,284***^1^* | **N = 3,265***^1^* |
| **Demographics** | | |
| Age | 59 (48, 72) | 65 (52, 77) |
| Time since CPO | 5 (2, 14) | 5 (2, 16) |
| **Cardiovascular risk factors, n (%)** | | |
| Previous CAD | 2,342 (37%) | 787 (24%) |
| Previous MI | 1,686 (27%) | 493 (15%) |
| Previous Revascularisation | 1,832 (29%) | 505 (15%) |
| Previous stroke | 268 (4.9%) | 161 (5.8%) |
| **History, n (%)** | | |
| Hypertension | 3,785 (60%) | 1,929 (59%) |
| Dyslipidemia | 2,794 (51%) | 1,206 (43%) |
| Diabetes | 1,183 (19%) | 554 (17%) |
| Active smoker | 2,103 (34%) | 711 (22%) |
| **ECG findings, n (%)** | | |
| T wave inversion | 4,676 (75%) | 2,462 (76%) |
| ST depression | 589 (9.5%) | 301 (9.3%) |
| Aberration (BBB, pacemaker) | 560 (9.0%) | 298 (9.2%) |
| None | 389 (6.3%) | 170 (5.3%) |
| **Medications at presentation, n (%)** | | |
| Anticoagulants | 622 (9.9%) | 376 (12%) |
| Beta-blockers | 2,160 (34%) | 1,043 (32%) |
| Statins | 2,044 (37%) | 799 (29%) |
| **Laboratory findings, median (IQR)** | | |
| eGFR | 88 (71, 102) | 84 (65, 98) |
| **Troponin, median (IQR)** | | |
| Hs-cTnT 0h | 9 (5, 24) | 7 (3, 18) |
| Hs-cTnT 1h | 9 (5, 24) | 7 (3, 18) |
| Hs-cTnT 2h | 9 (5, 26) | 7 (3, 19) |
| Hs-cTnT 3h | 12 (6, 35) | 9 (4, 26) |
| First consecutive Hs-cTnT | 9 (5, 25) | 7 (3, 18) |
| *^1^*Median (Q1, Q3); n (%)CAD = coronary artery disease; MI = myocardial infarction; ECG = Electrocardiogram; BBB=Bundle branch block; eGFR = estimated glomerular filtration rate; ACEIs/ARBs = Angiotensin-converting enzyme inhibitors or angiotensin receptor blocker; hs-cTnT = high-sensitivity cardiac troponin T | | |

**Supplemental Figures**

**Supplemental Figure 1.**

**
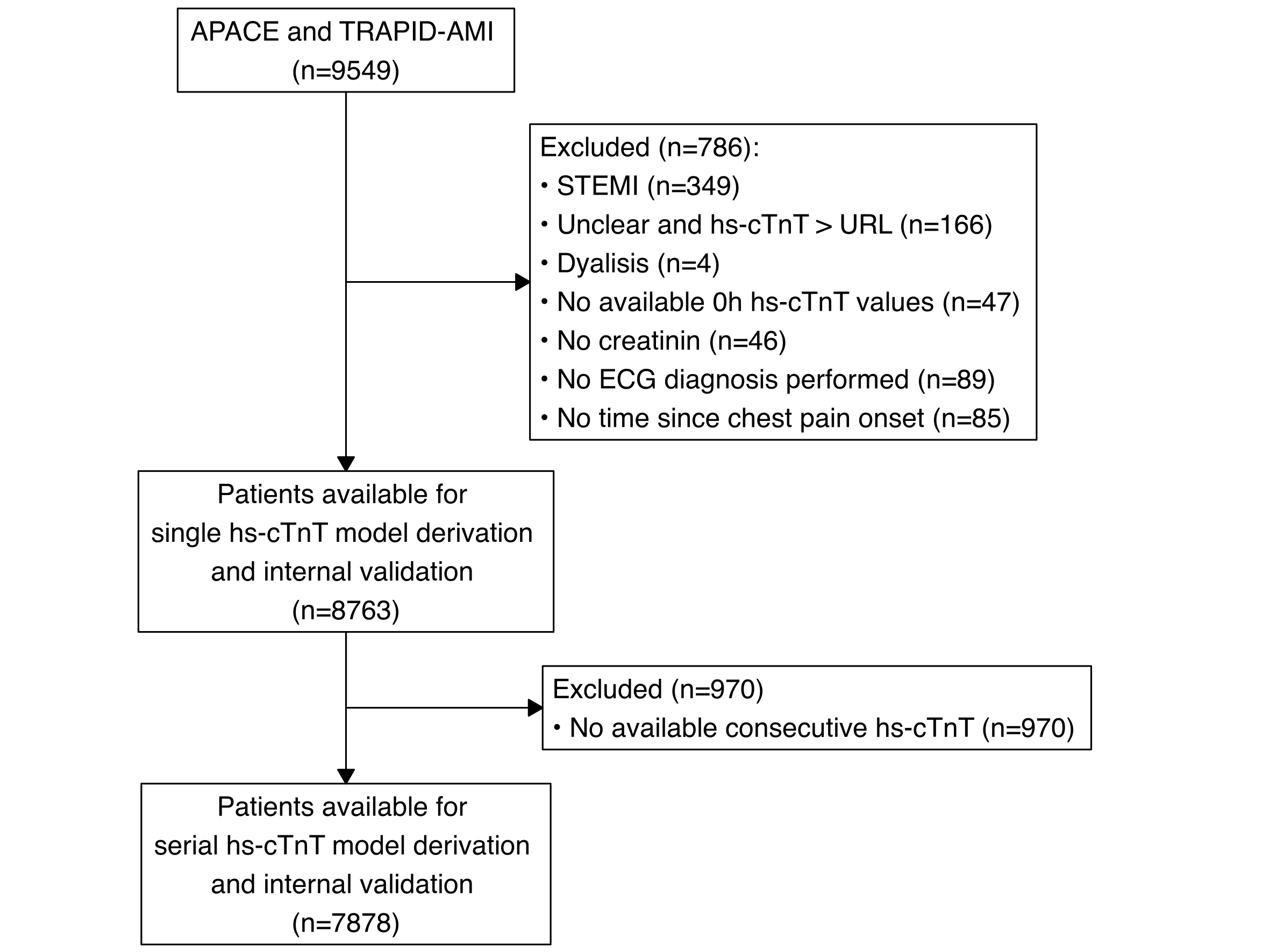
**

**Supplemental Figure 2.**

**
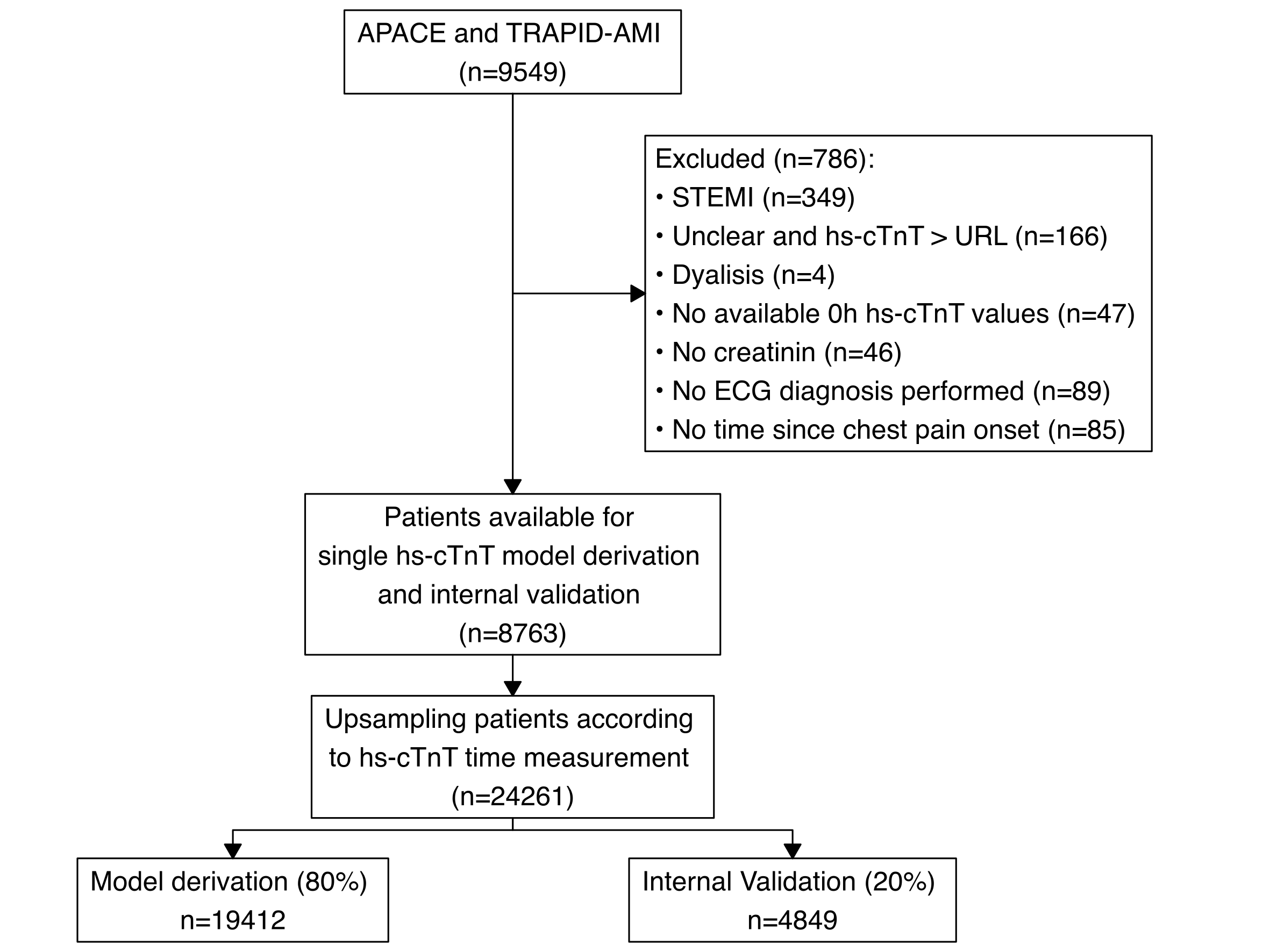
**

**Supplemental Figure 3**

**
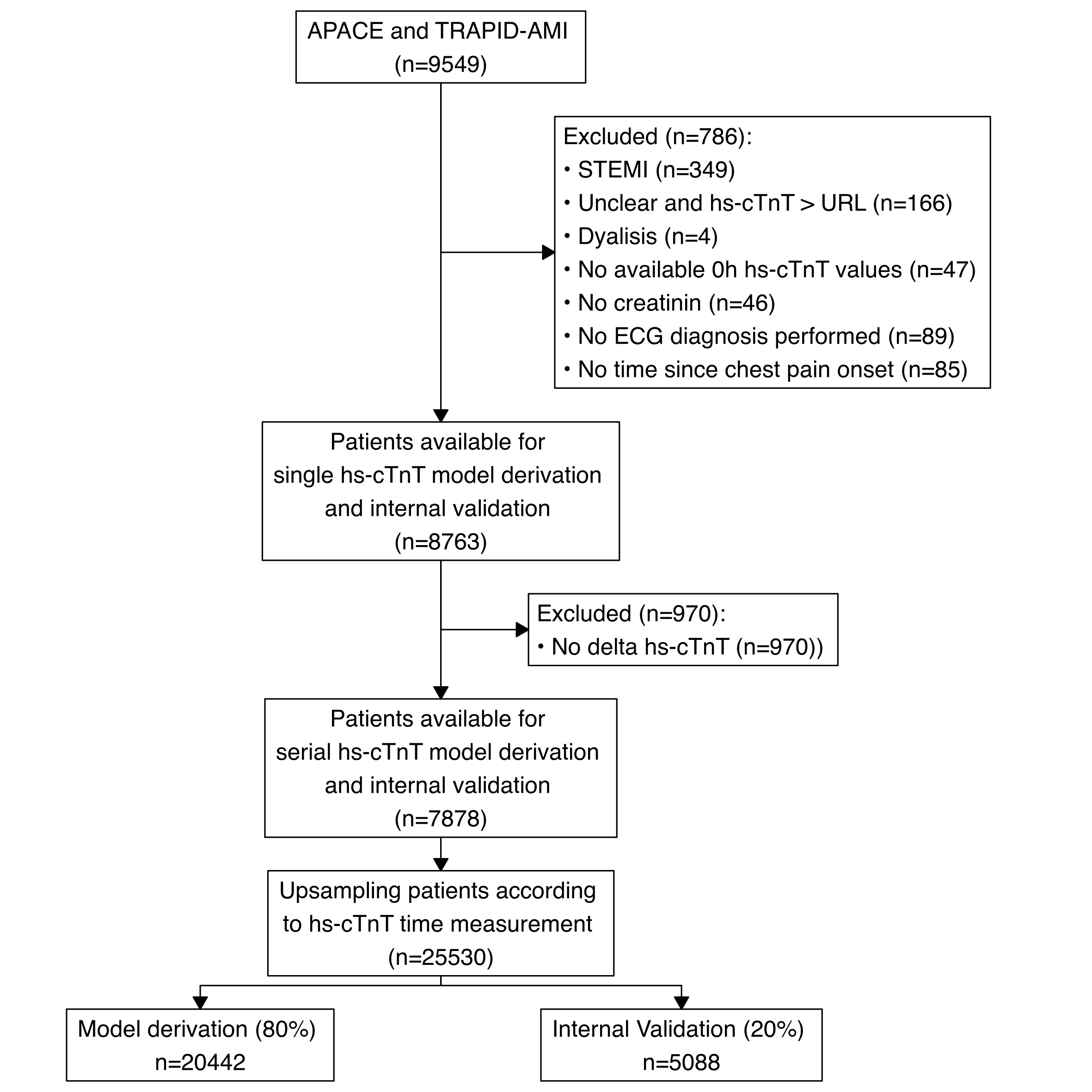
**

**Supplemental Figure 4.**

**
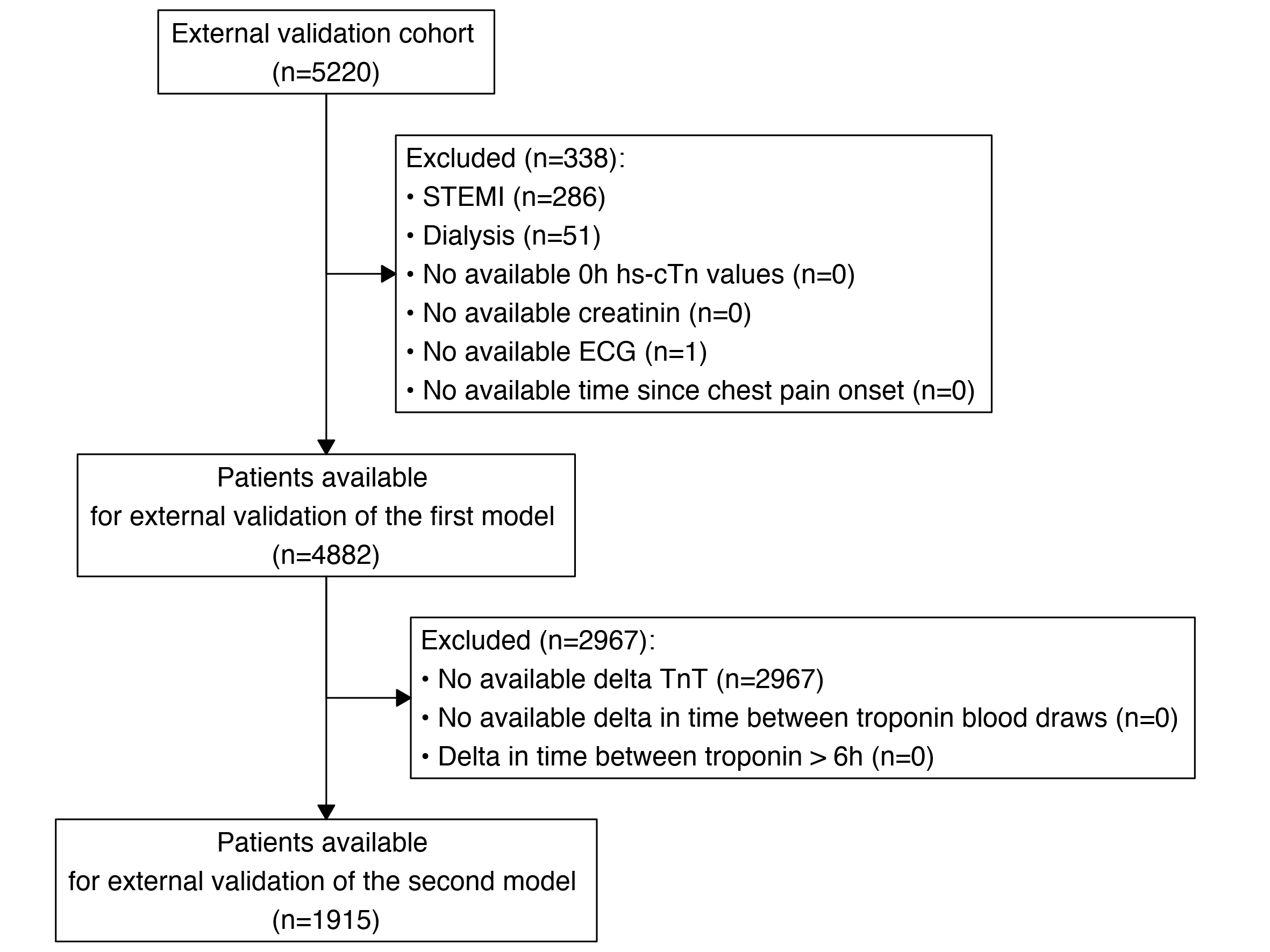
**

**
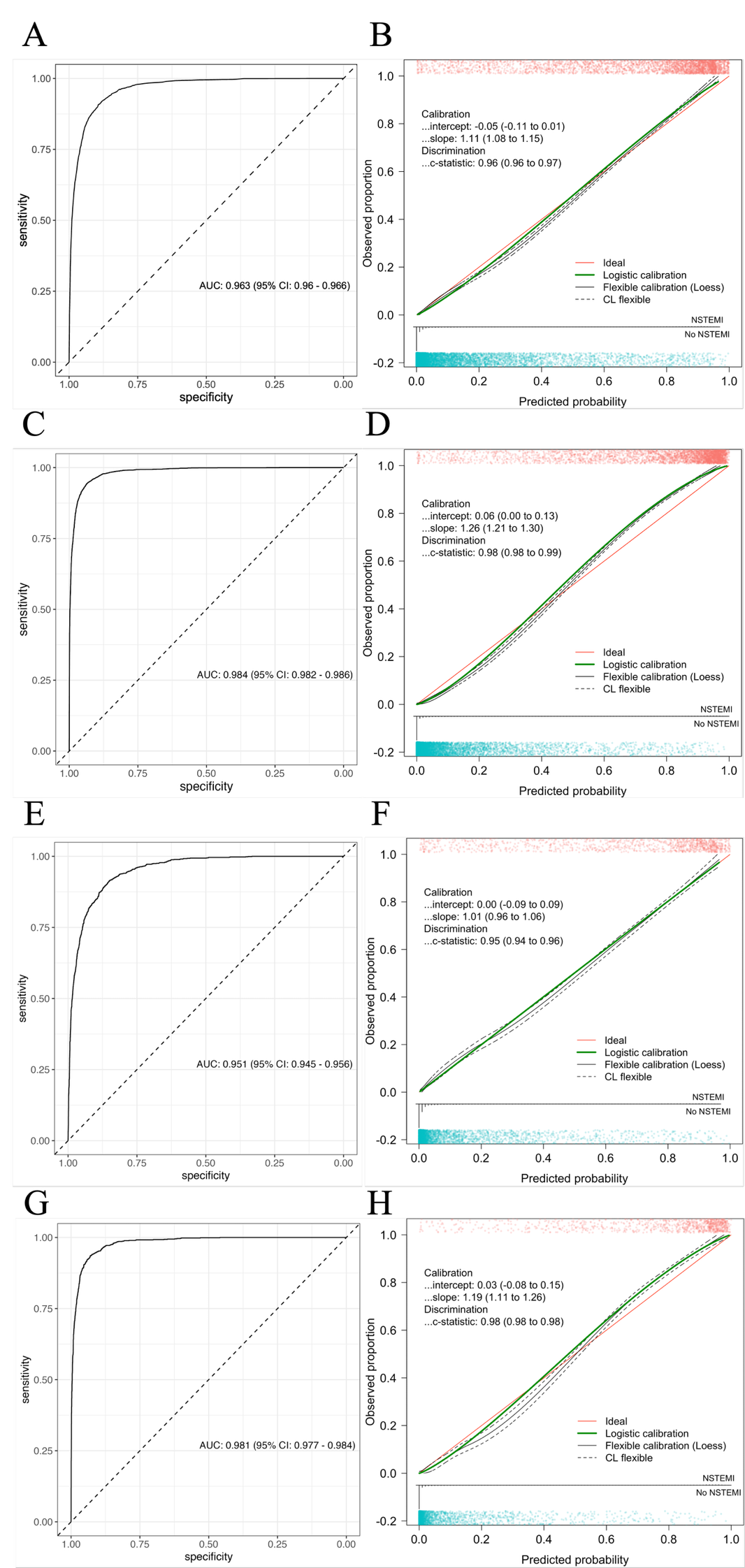
Supplemental Figure 5**

**
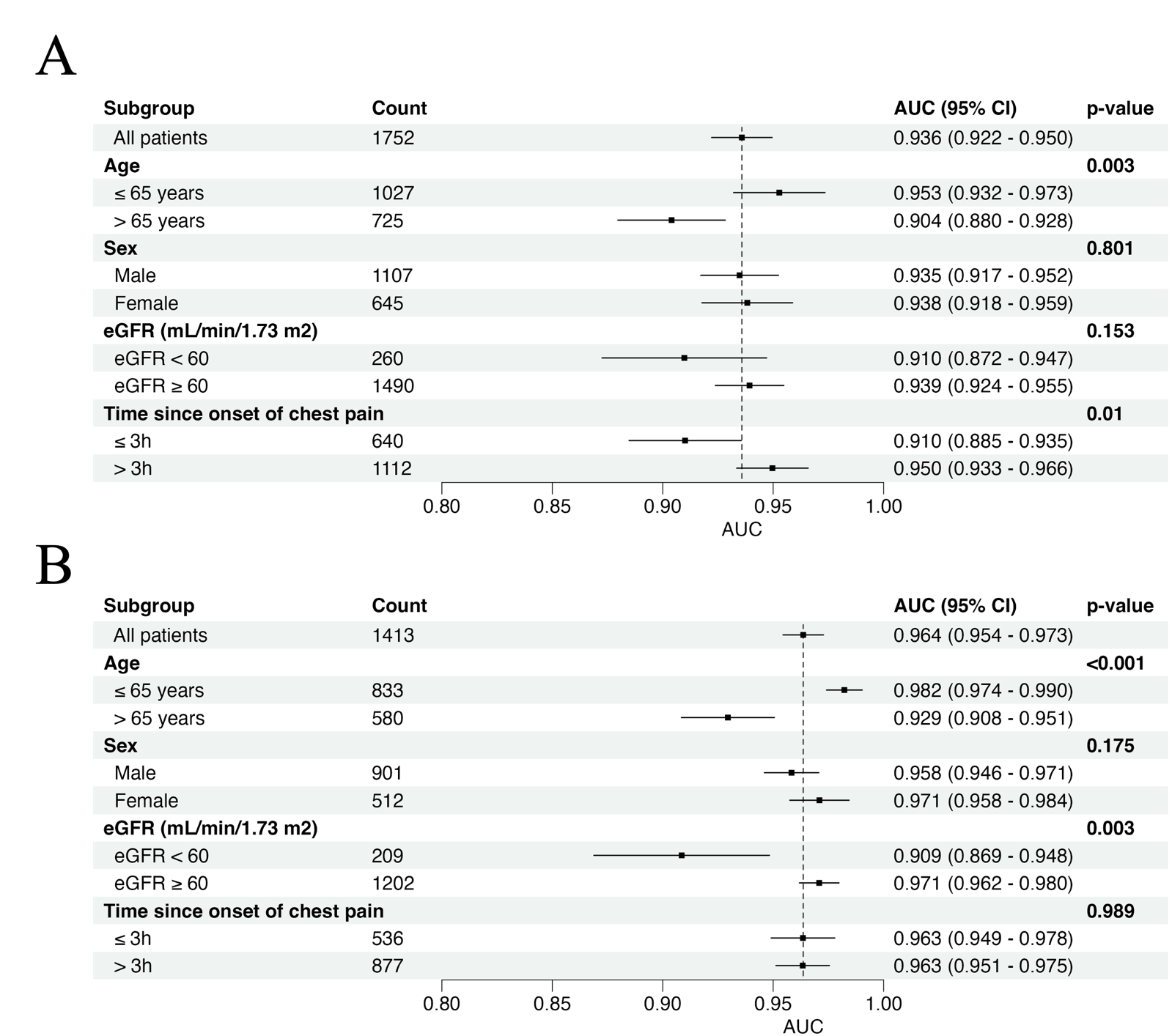
Supplemental Figure 6**

**
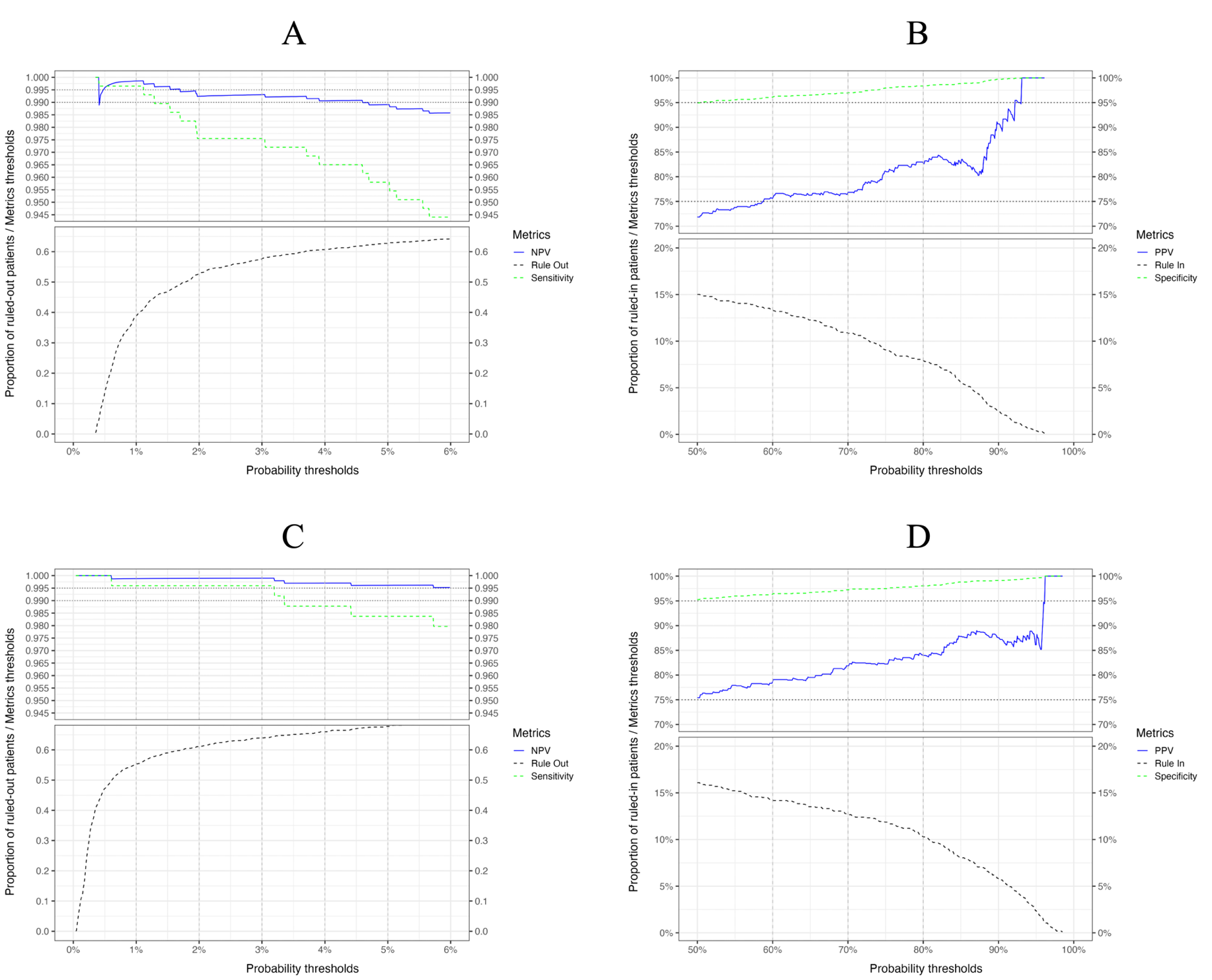
Supplemental Figure 7.**

**
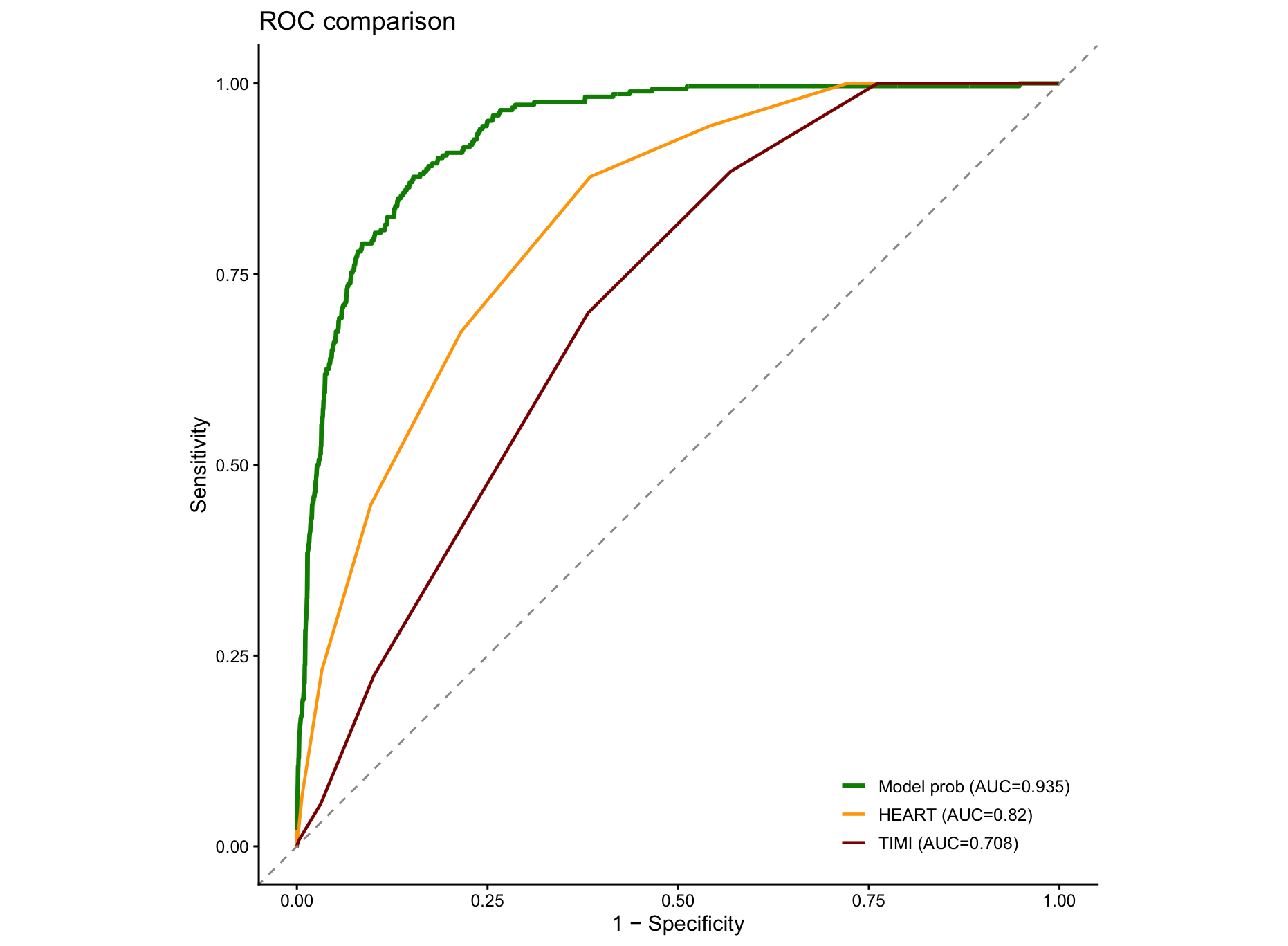
 Supplemental Figure 8.**

**
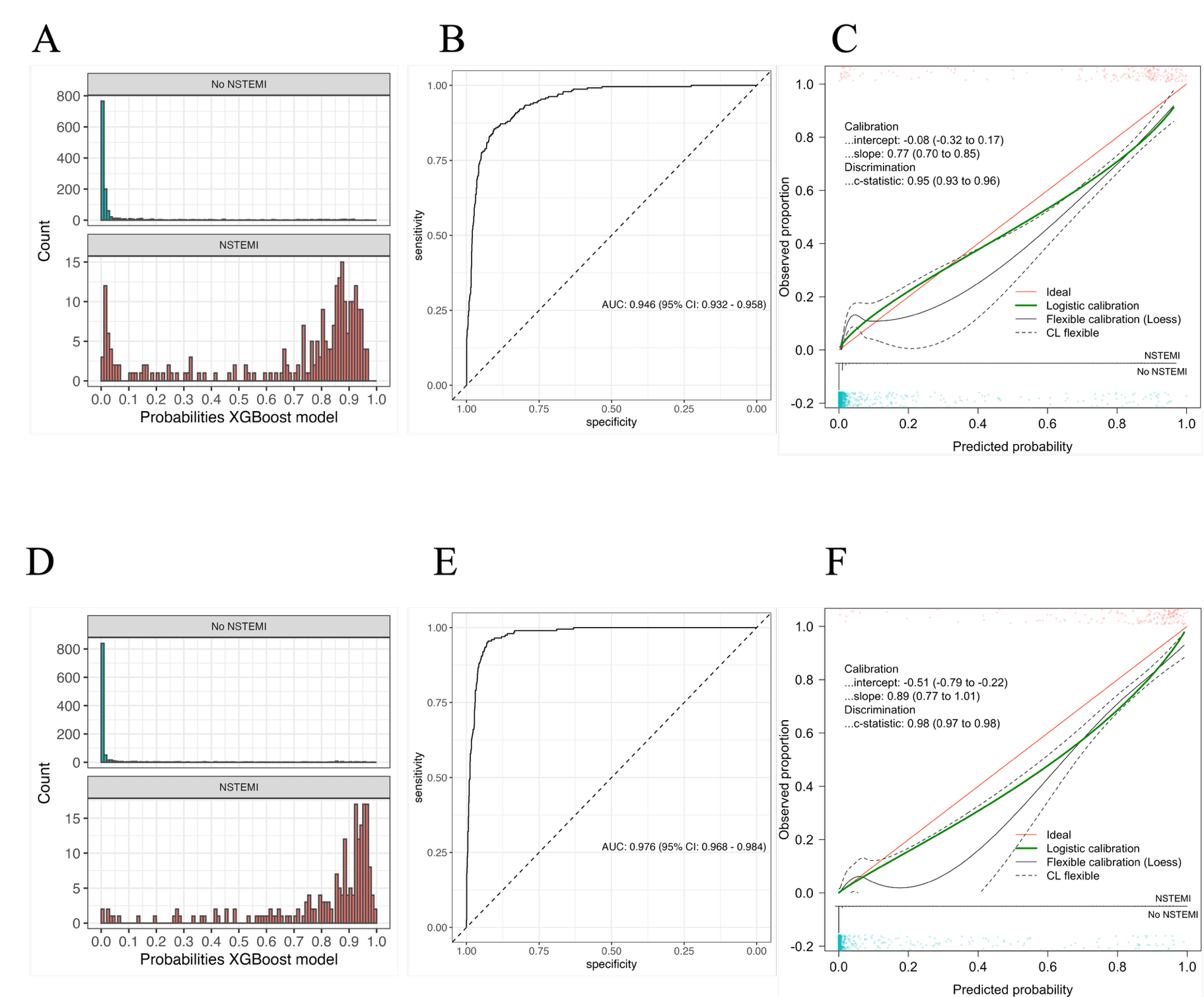
Supplemental Figure 9.**

**
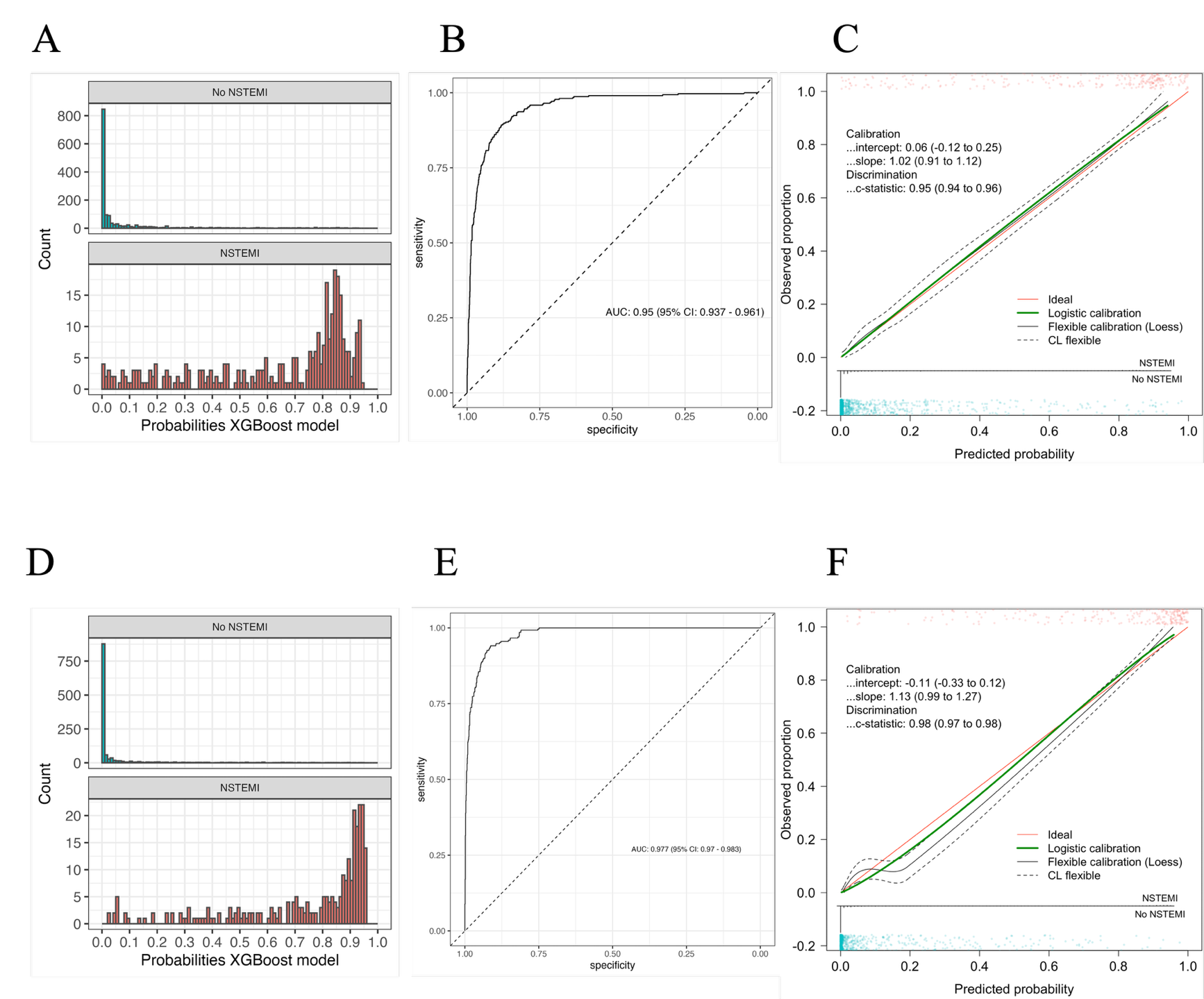
Supplemental Figure 10.**

**Supplemental Figure 11.**

**
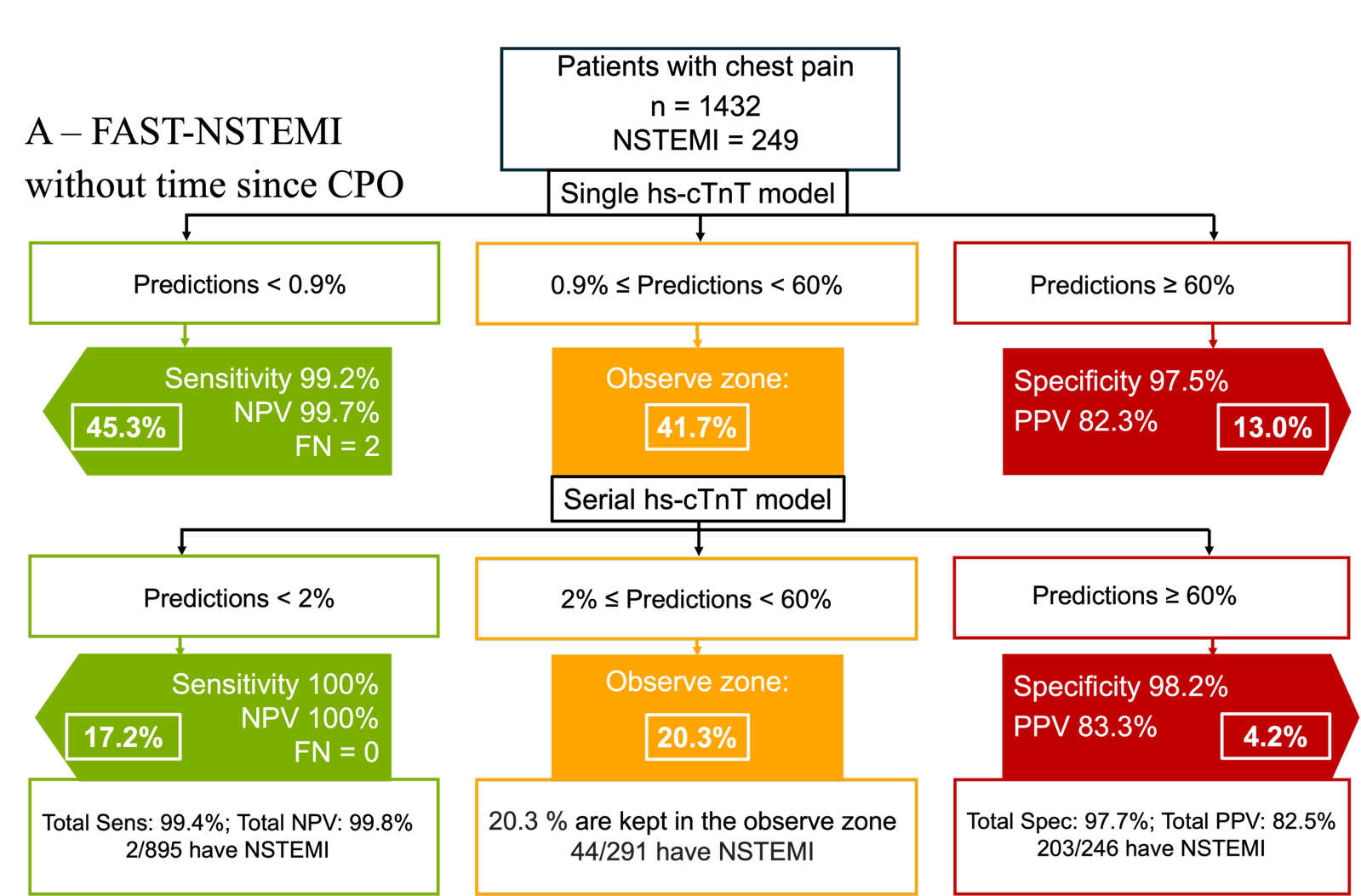
**

**
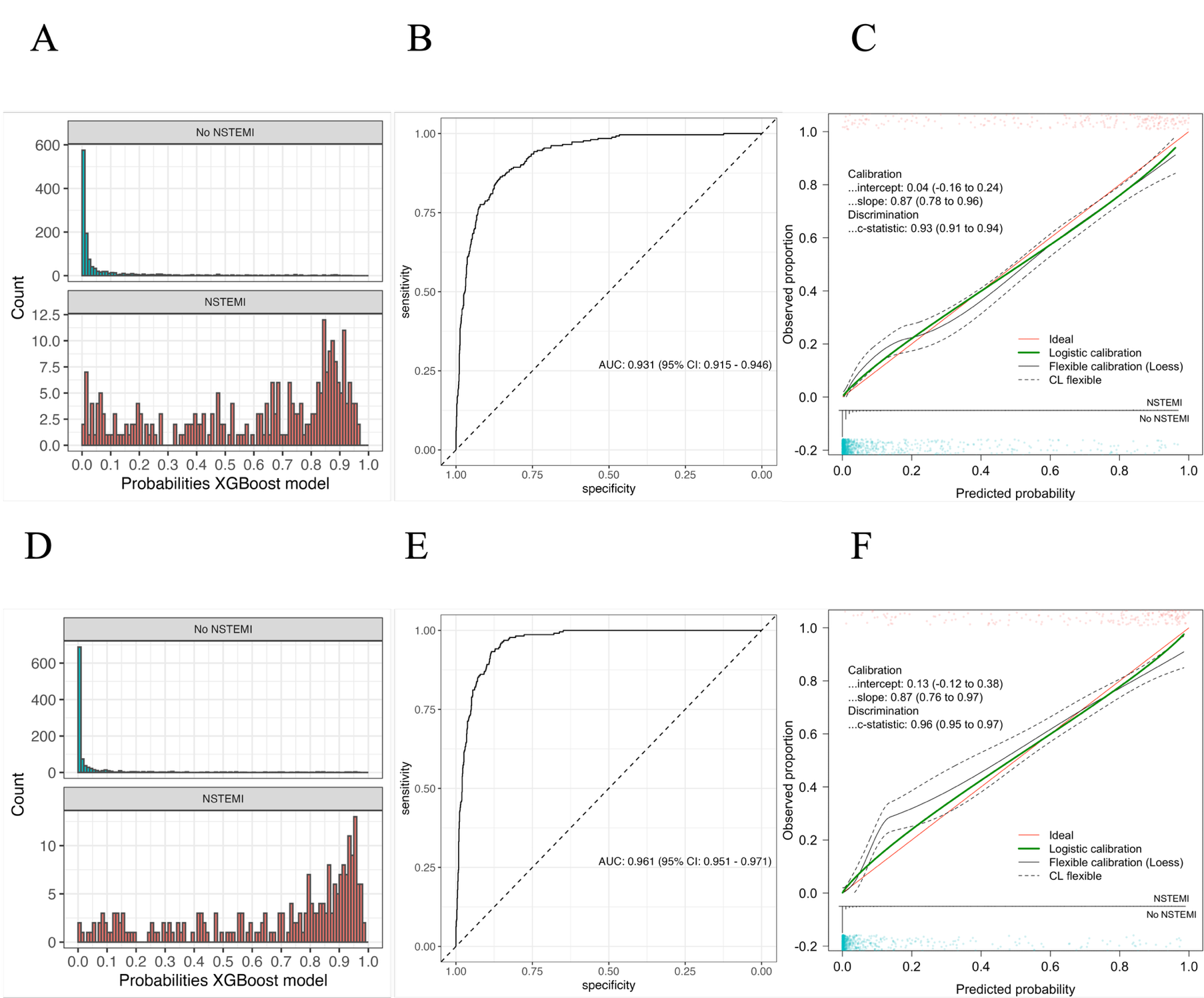
Supplemental Figure 12.**

**
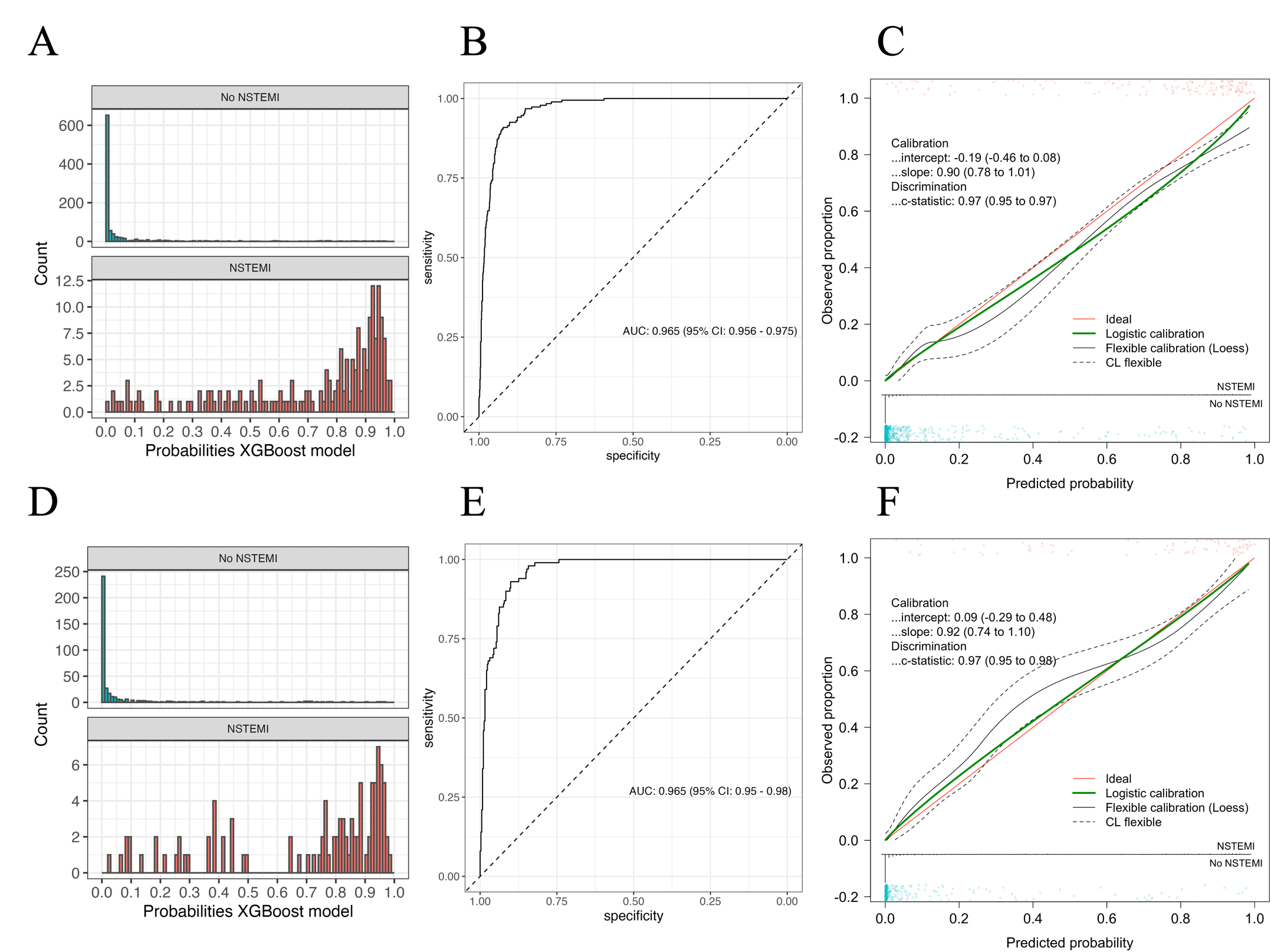
Supplemental Figure 13.**

**Supplemental Figure 14.**

**
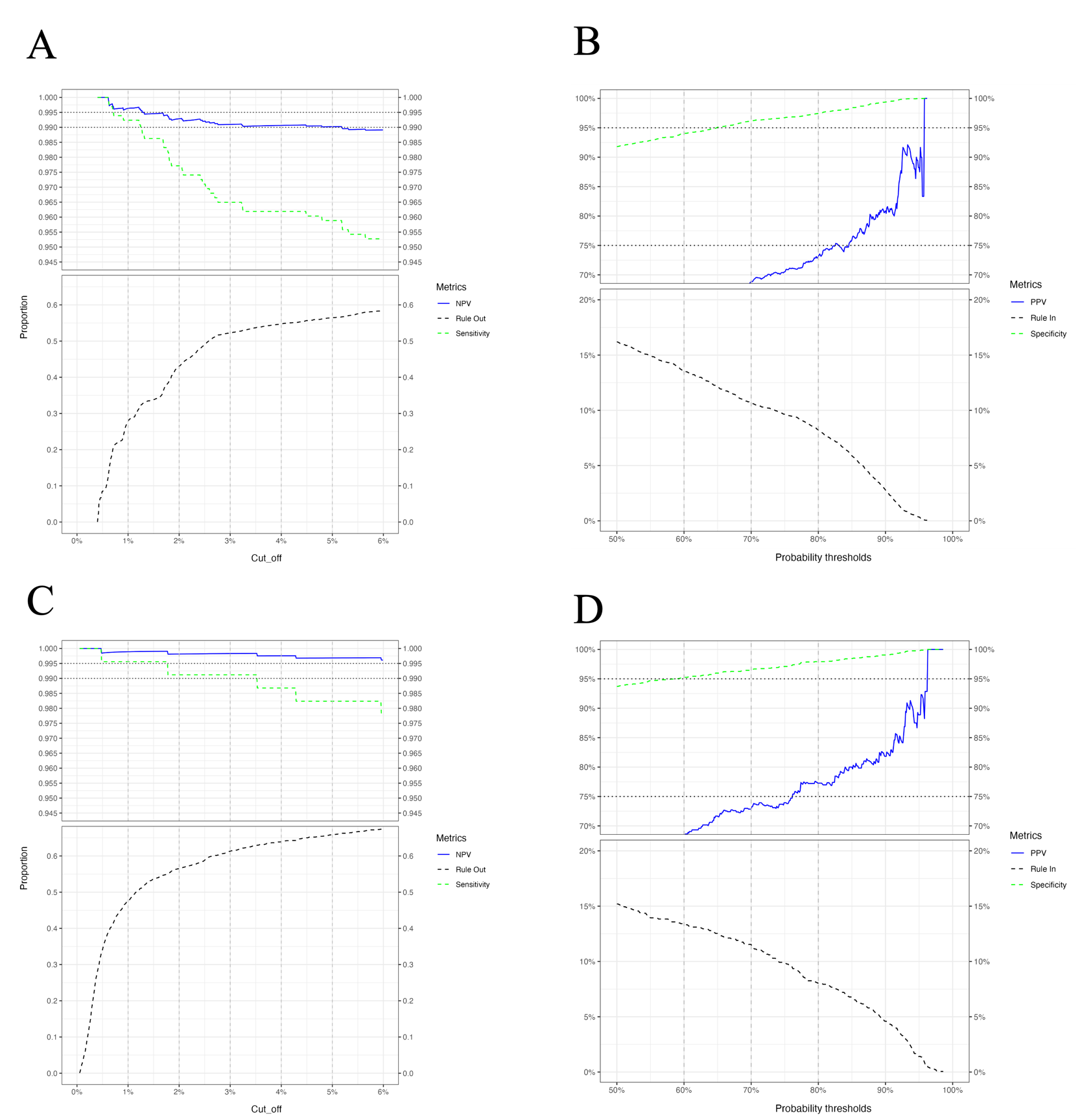
**

**
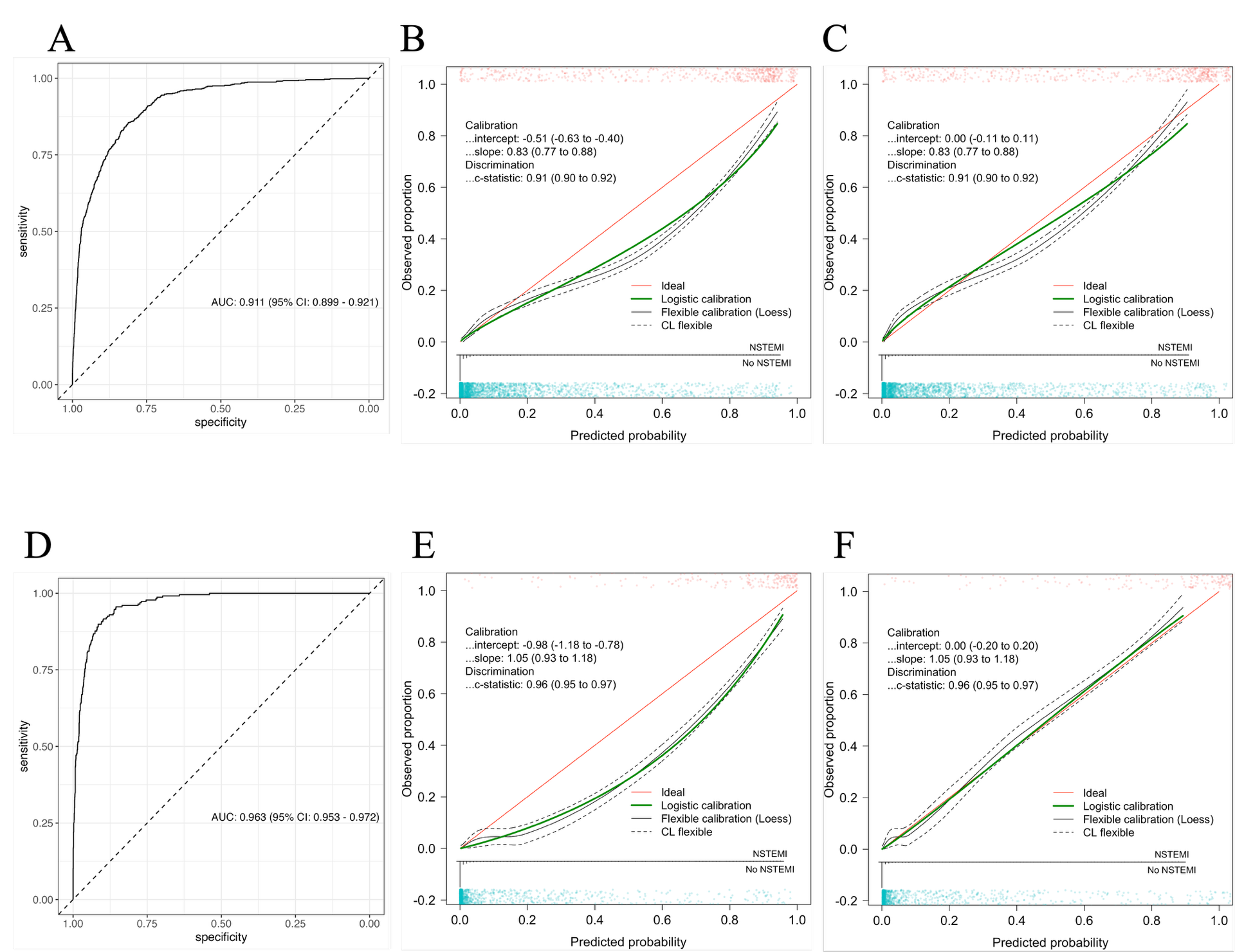
Supplemental Figure 15.**

**Supplemental Figure 16.**

**
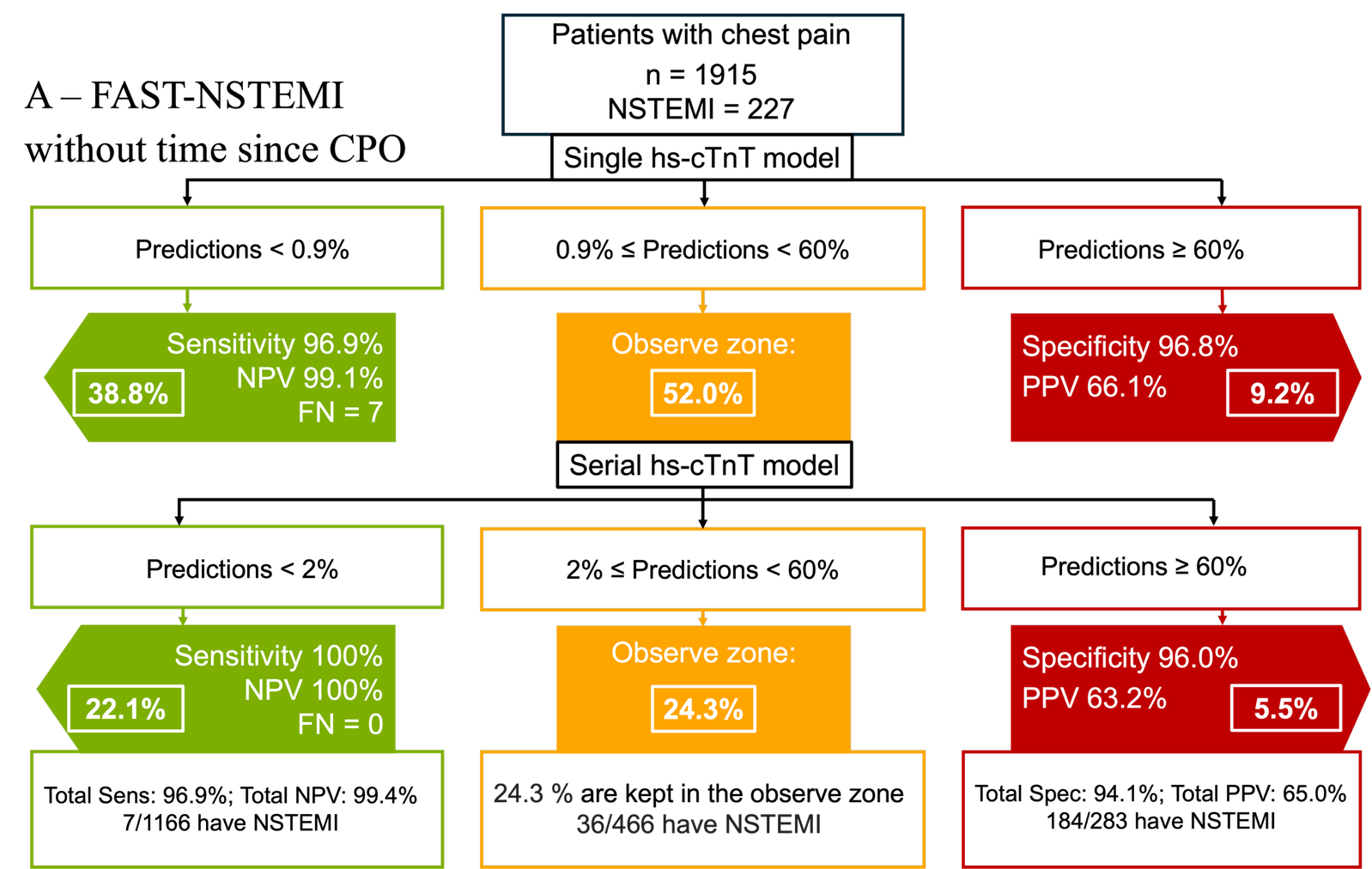
**

**Supplemental Figure 17.**

**
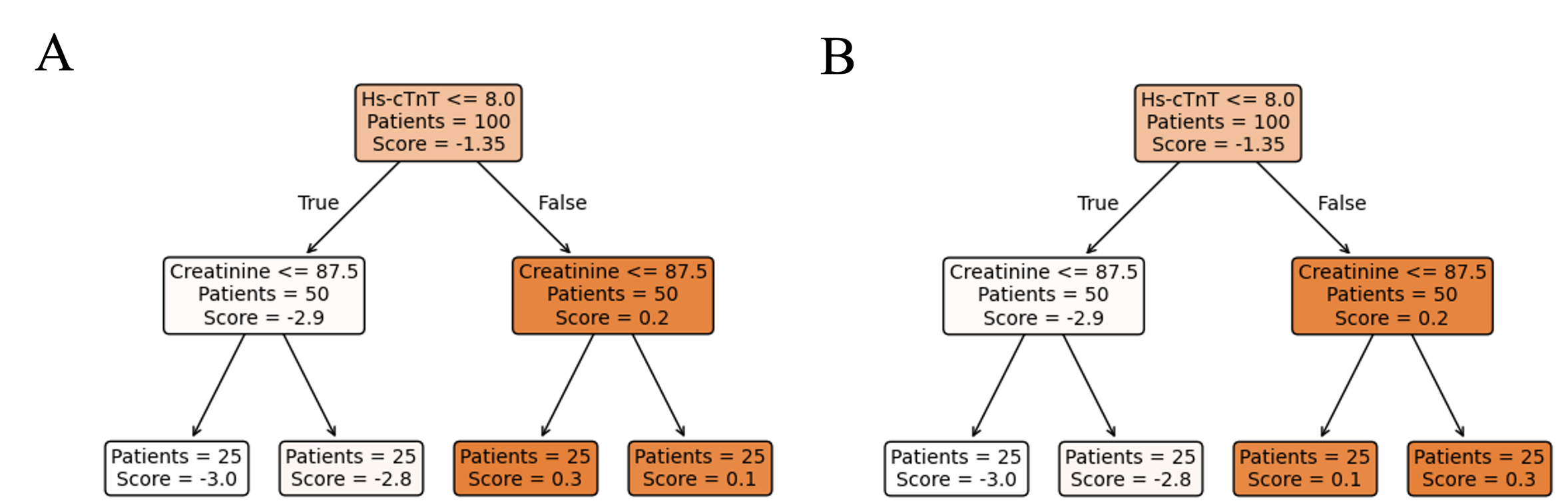
**

**Supplemental Figure 18.**

**
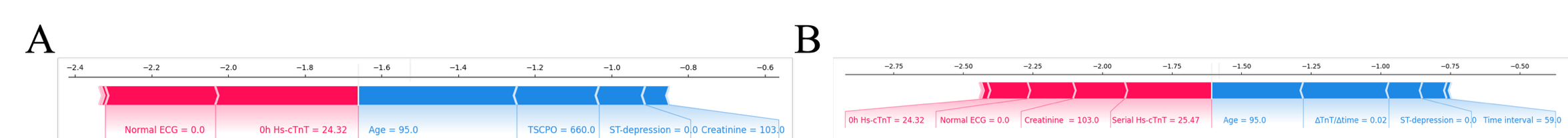
**

**
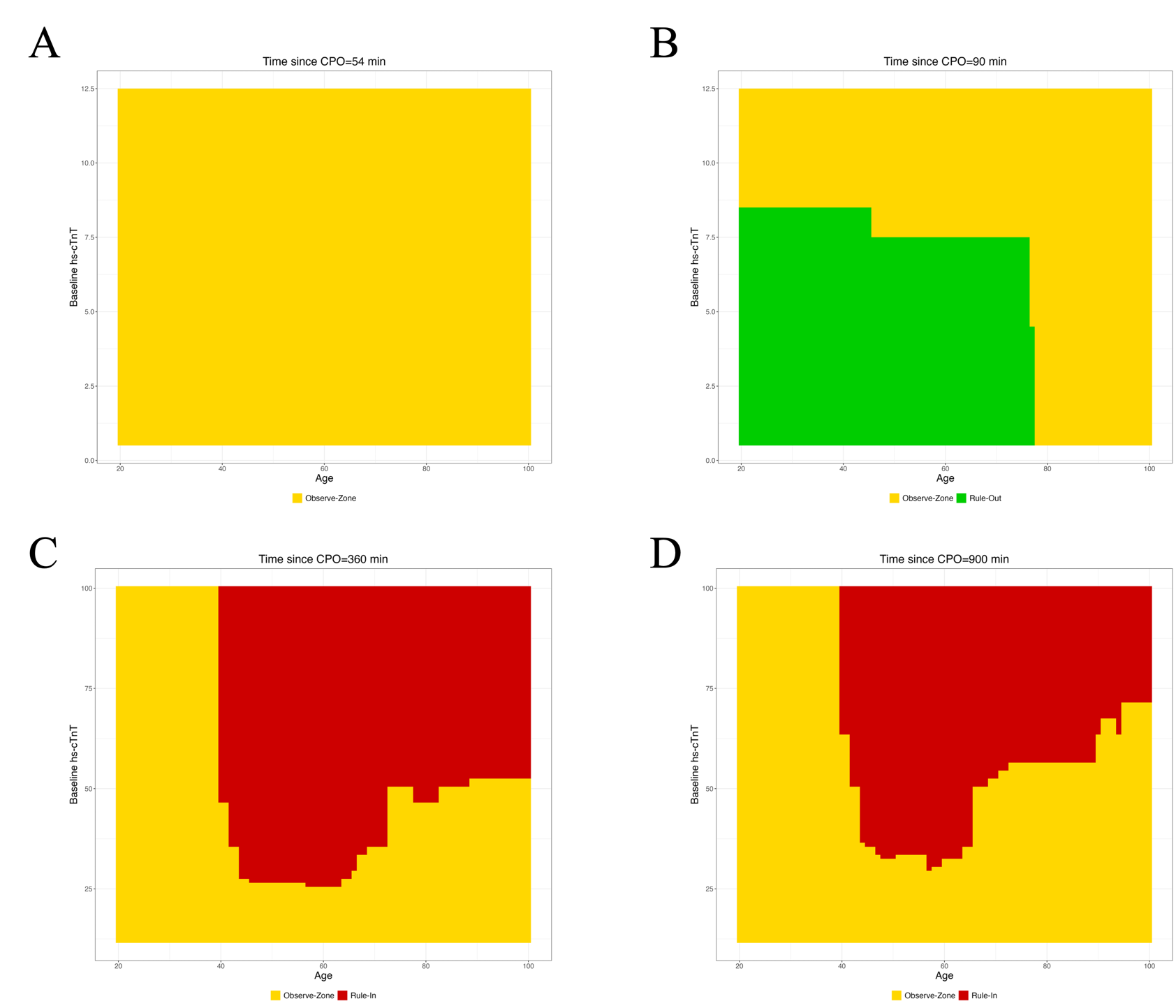
Supplemental Figure 19.**
